# StSIRT1 regulates potato dormancy through deacetylation of peroxidase-like 3 at K37

**DOI:** 10.1126/sciadv.aef7735

**Published:** 2026-10-28

**Authors:** Qiannan Hu, Yingying Dong, Hongxia Hu, Yizhou Xiang, Bailin Liu, Tuck-Whye Wong, Zhaojun Ban, Zisheng Luo, Li Li

**Affiliations:** ^1^ College of Biosystems Engineering and Food Science, Zhejiang University, Hangzhou 310058, China.; ^2^ Key Laboratory of Agro-Products Postharvest Handling, Ministry of Agriculture and Rural Affairs, Zhejiang Key Laboratory of Agri-food Resources and High-value Utilization, Innovation Centre for Postharvest Agro-Products Technology, Zhejiang University, Hangzhou 310058, China.; ^3^ State Key Laboratory for Crop Stress Resistance and High-Efficiency Production, College of Agronomy, Northwest A&F University, Yangling 712100, China.; ^4^ Sustainable and Smart Materials Laboratory, Department of Biomedical Engineering and Health Sciences, Universiti Teknologi Malaysia, Johor Bahru 81310, Malaysia.; ^5^ Key Laboratory of Chemical and Biological Processing Technology for Farm Products of Zhejiang Province, Zhejiang Provincial Collaborative Innovation Centre of Agricultural Biological Resources Biochemical Manufacturing, School of Biological and Chemical Engineering, Zhejiang University of Science and Technology, Hangzhou 310023, China.

## Abstract

Plants have evolved sophisticated defense systems to synchronize growth with environmental fluctuations, including the adaptive strategy of dormancy, an important agronomic trait. Nevertheless, the role of posttranslational modification of nonhistone proteins in dormancy regulation remains elusive. Here, we report that sirtuin (StSIRT1)–mediated deacetylation of peroxidase (StPRX3) suppresses peroxidase activity, culminating in potato (*Solanum tuberosum* L.) dormancy release. The lysine deacetylase StSIRT1—suppressed by the dormancy regulator nicotinamide (NAM)—accumulates during dormancy release and negatively modulates StPRX3. Furthermore, lysine-37 (K37; K denotes lysine) in StPRX3 is essential for its direct interaction with StSIRT1. StSIRT1 interacts with StPRX3 to regulate the hydrogen peroxide (H_2_O_2_)–scavenging capacity of peroxidase by decreasing K37 acetylation levels. Notably, the StPRX3^K37R^ (K37→arginine, K37R; R denotes arginine) mutant suppresses peroxidase activity, leading to accelerated potato dormancy release. Our findings uncover an StSIRT1-StPRX3 cascade in dormancy regulation that activates peroxidase and reduces H_2_O_2_ levels in potato, providing insights into the complex regulatory networks involved in dormancy.

## INTRODUCTION

Plants have evolved sophisticated strategies to synchronize growth with environmental fluctuations (*1*). Higher plants use dormancy, an adaptive developmental arrest, to survive unfavorable environmental conditions, such as cold, drought, nutrient limitation, and mechanical injury, and to resume growth when conditions improve (*2*–*4*). In geophytic species (e.g., potato, yam, onion, and tulip), dormancy and subsequent sprouting determine postharvest stability, yield potential, and market availability (*5*). Given that controlled dormancy prolongs storage life and timely release from dormancy ensures uniform sprouting and optimal productivity, dormancy regulation is crucial for supply chain stability and global food security (*5*, *6*). Despite the agronomic importance of dormancy, the molecular mechanisms through which plants perceive and integrate environmental cues to initiate dormancy and its subsequent release remain poorly understood.

Dormancy transitions are controlled by complex signaling networks in which reactive oxygen species (ROS) serve as central regulators of cellular redox balance and developmental reactivation (*7*, *8*). ROS, predominantly produced through mitochondrial respiration and nicotinamide adenine dinucleotide phosphate (NADPH) oxidase activity, generate a transient oxidative window during dormancy release that facilitates lipid peroxidation, protein turnover, and mitochondrial restructuring, thereby triggering sprouting (*9*). ROS accumulation also induces mRNA oxidation, microRNA oxidation, and protein carbonylation, processes associated with dormancy release (*10*). In addition, ROS-induced cell wall loosening supports bud emergence and elongation (*9*). Because of its relative stability and diffusibility, hydrogen peroxide (H_2_O_2_) functions as a key signaling molecule among ROS (*11*, *12*). It integrates environmental and hormonal cues by promoting abscisic acid catabolism and stimulating gibberellin biosynthesis, shifting dormancy toward sprouting (*7*, *13*). Despite increasing evidence of ROS involvement in dormancy control, the mechanisms underlying the precise modulation of ROS production and detoxification required to fine-tune dormancy release remain unclear.

Lysine acetylation is a conserved, reversible posttranslational modification that dynamically regulates plant development and stress adaptation (*14*). There is evidence that lysine (K) acetylation may regulate plant dormancy and sprouting by altering chromatin accessibility and metabolic activity (*15*–*17*). Histone acetylation changes dynamically during dormancy transitions; for instance, acetylation of histones 2A (H2A) and H2B increases during bud dormancy release in poplar (*Populus tremula × Populus alba*) (*18*). Similarly, acetylation of H3K18, H4K16, and H4K5 in alfalfa (*Oryza sativa* L.) seeds peaks during imbibition and declines upon sprouting (*19*). The discovery of nonhistone acetylation has expanded the functional scope of lysine acetylation beyond chromatin dynamics and transcriptional regulation to include protein stability, enzymatic activity, subcellular localization, and protein-protein interactions (*20*, *21*). Although the regulatory roles of nonhistone acetylation in plants are increasingly recognized, the molecular mechanisms by which nonhistone lysine acetylation orchestrates dormancy transitions remain elusive.

We previously showed that the deacetylase inhibitor nicotinamide (NAM) prolongs potato dormancy by maintaining redox homeostasis and limiting H_2_O_2_ accumulation (*22*). However, it has remained unclear whether NAM acts via modulation of protein acetylation. Here, we identified the sirtuin (SIRT)–type deacetylase StSIRT1 as a key regulator of potato dormancy release; its expression is repressed by NAM. Proteomic analysis characterized StPRX3 as a NAM-responsive substrate of StSIRT1, which directly interacts with and deacetylates StPRX3 at K37, thereby reducing peroxidase activity, increasing H_2_O_2_ accumulation, and promoting dormancy release. These findings reveal an StSIRT1-StPRX3-H_2_O_2_ regulatory module that links deacetylation with ROS metabolism and provides previously unidentified insight into the interplay between redox signaling and posttranslational modification in the control of plant dormancy.

## RESULTS

### The deacetylase gene *StSIRT1* is negatively associated with dormancy maintenance

Previous work showed that exogenous NAM extended potato dormancy by stabilizing cellular redox balance and reducing H_2_O_2_ accumulation (*22*). In the present study, we found that NAM continued to suppress bud development even at 24 to 48 days (Fig. 1A). Although sprouting rate reflects the overall effect of NAM, temporal dynamics in bud growth further support its sustained inhibitory effect (Fig. 1C). We collected buds (≥2 mm) from 6 to 24 days and observed that size differences between NAM-treated and control tubers emerged from 18 days onward, suggesting that internal physiological processes may have initiated earlier (Fig. 1B and fig. S1A). NAM-treated potatoes exhibited a 50% reduction in solanine content at 12 days and altered transcription of canonical dormancy markers (*StDOG1*, *StABI5*, and *StCYP707A1*) and ROS-producing respiratory burst oxidase homolog (RBOH) genes (*StRBOHA* and *StRBOHB*) (Fig. 1, A to E). Given that NAM is a potent inhibitor of SIRT-type deacetylases, we examined whether its dormancy-prolonging effect was mediated by a specific SIRT (*23*). Phylogenetic analysis identified two SIRT homologs in potato, StSIRT1 and StSIRT2. StSIRT1 clustered closely with AtSIRT8, whereas StSIRT2 grouped within the same major clade but separated from StSIRT1, suggesting evolutionary divergence between the two SIRT homologs (Fig. 1F). We compared the temporal and spatial expression patterns of two potato *SIRT* homologs using quantitative reverse transcription polymerase chain reaction (qRT-PCR). The results showed that *StSIRT1* progressively was up-regulated during dormancy release, with a 4.6-fold increase at the onset of visible sprouting. NAM treatment suppressed *StSIRT1* expression from 12 days onward, indicating that *StSIRT1* functions mainly during the dormancy release phase and is NAM sensitive (Fig. 1G). In contrast, *StSIRT2* showed low expression during storage and was unresponsive to NAM (Fig. 1I). To confirm that *StSIRT1* responds specifically to NAM, we treated potatoes with sirtinol, a SIRT inhibitor. Sirtinol treatment induced no phenotypic changes or *StSIRT1* response relative to the control (fig. S2). Tissue-specific heatmap analysis showed that *StSIRT1* exhibited the highest expression in bud meristems and the pulp beneath buds, especially during dormancy breaking, suggesting a close association with sprouting, whereas *StSIRT2* was mainly enriched in the peel (Fig. 1, H and J).

**Fig. 1.**
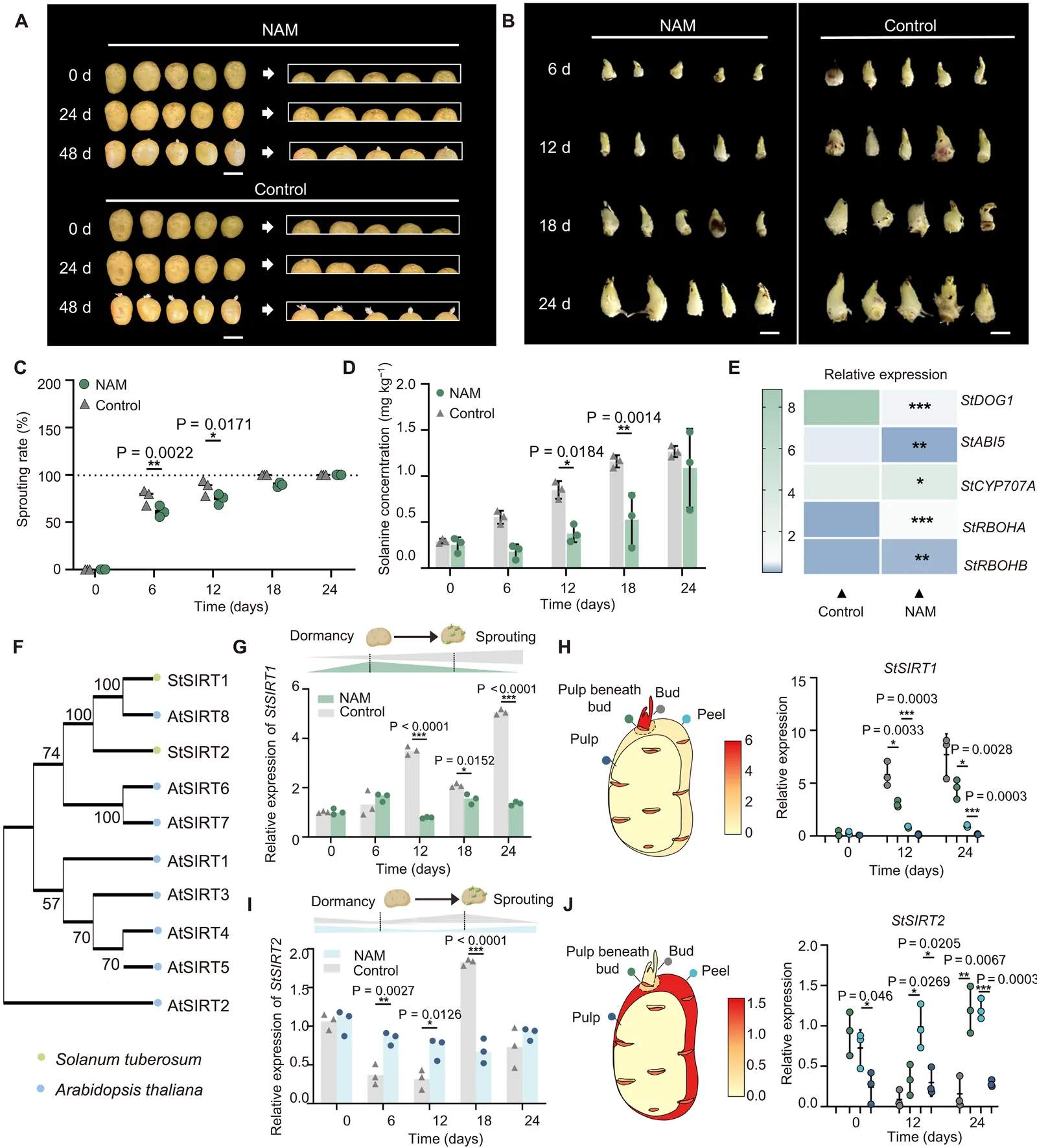
NAM prolongs dormancy by suppressing *StSIRT1* in potato. (**A**) Phenotypes of potatoes treated with NAM (300 mM). Potatoes were stored under dark conditions at 20°C for 48 days, with images recorded every 12 days. Scale bars, 5 cm. d, days. (**B**) Phenotypes of potato buds, with images recorded every 6 days. Scale bars, 1 cm. (**C** and **D**) Sprouting rate and solanine concentrations of NAM-treated potatoes. (**E**) Relative transcript levels of *StDOG1*, *StABI5*, *StCYP707A*, *StRBOHA*, and *StRBOHB* in NAM-treated potatoes; *EF-1*α was used as an internal control. (**F**) Phylogenetic analysis of the SIRT family in *Solanum tuberosum* L. (**G** and **I**) Expression levels and patterns of *StSIRT1*and *StSIRT2* in response to NAM in potatoes; *EF-1*α was used as an internal control. (**H** and **J**) Tissue-specific heatmap at 12 days and spatiotemporal expression of *StSIRT1* and *StSIRT2* in bud, peel, pulp, and pulp beneath bud; *EF-1*α was used as an internal control. Values are means ± SE; *n* = 5 for (A) and (B) and *n* = 3 for [(C) to (E) and (G) to (J)]. Two-way analysis of variance (ANOVA) with Tukey’s multiple-comparison test was used in [(C) and (D) and (G) to (J)]; one-way ANOVA with Tukey’s test was used in (E). *P < 0.05, **P < 0.01, and ***P < 0.001. [(H) and (J)] created in BioRender. Li, L. (2026) https://BioRender.com/1nhkapo, and were licensed under CC BY 4.0.

To elucidate the role of StSIRT1 in dormancy, we examined its sequence structural features and promoter (fig. S3). StSIRT1 is located within a 1733–base pair (bp) genomic fragment containing a 1416-bp coding sequence that encodes a 471–amino-acid protein with a predicted molecular mass of 52.53 kDa. Bioinformatic analyses predicted that StSIRT1 has a balanced hydropathicity profile and lacks transmembrane domains and signal peptide. Promoter analysis identified multiple dormancy-responsive cis-elements, including abscisic acid and gibberellin motifs, suggesting involvement in dormancy regulation. For functional validation of *StSIRT1*, RNA interference (*StSIRT1*-RNAi) and overexpression (*StSIRT1*-OE) were transiently performed to examine the effects of altered *StSIRT1* expression in potato. Overexpression of *StSIRT1* greatly accelerated sprouting, whereas RNAi-mediated silencing delayed dormancy release, confirming that *StSIRT1* negatively regulates dormancy (Fig. 2, A and B). qRT-PCR analysis confirmed robust modulation of *StSIRT1* gene expression, with a 13-fold increase in overexpression lines relative to empty-vector (EV) controls and reduction to ∼4% of control levels in RNAi potatoes (Fig. 2C). In StSIRT1-OE potatoes, transcript levels of *StDOG1* and *StNCED1* were reduced by ∼90% compared with EV potatoes, whereas *StCYP707A1* expression was 1.5-fold higher (Fig. 2, D to F). Conversely, StSIRT1-silenced potatoes exhibited substantially reduced *StCYP707A1* levels and elevated expression of *StDOG1* and *StNCED1*. Similar regulatory patterns were observed in transiently transformed tobacco leaves, where overexpression or silencing of *NbStSIRT1* altered the expression of *NbDOG1*, *NbNCED1*, and *NbCYP707A1* (fig. S4).

**Fig. 2.**
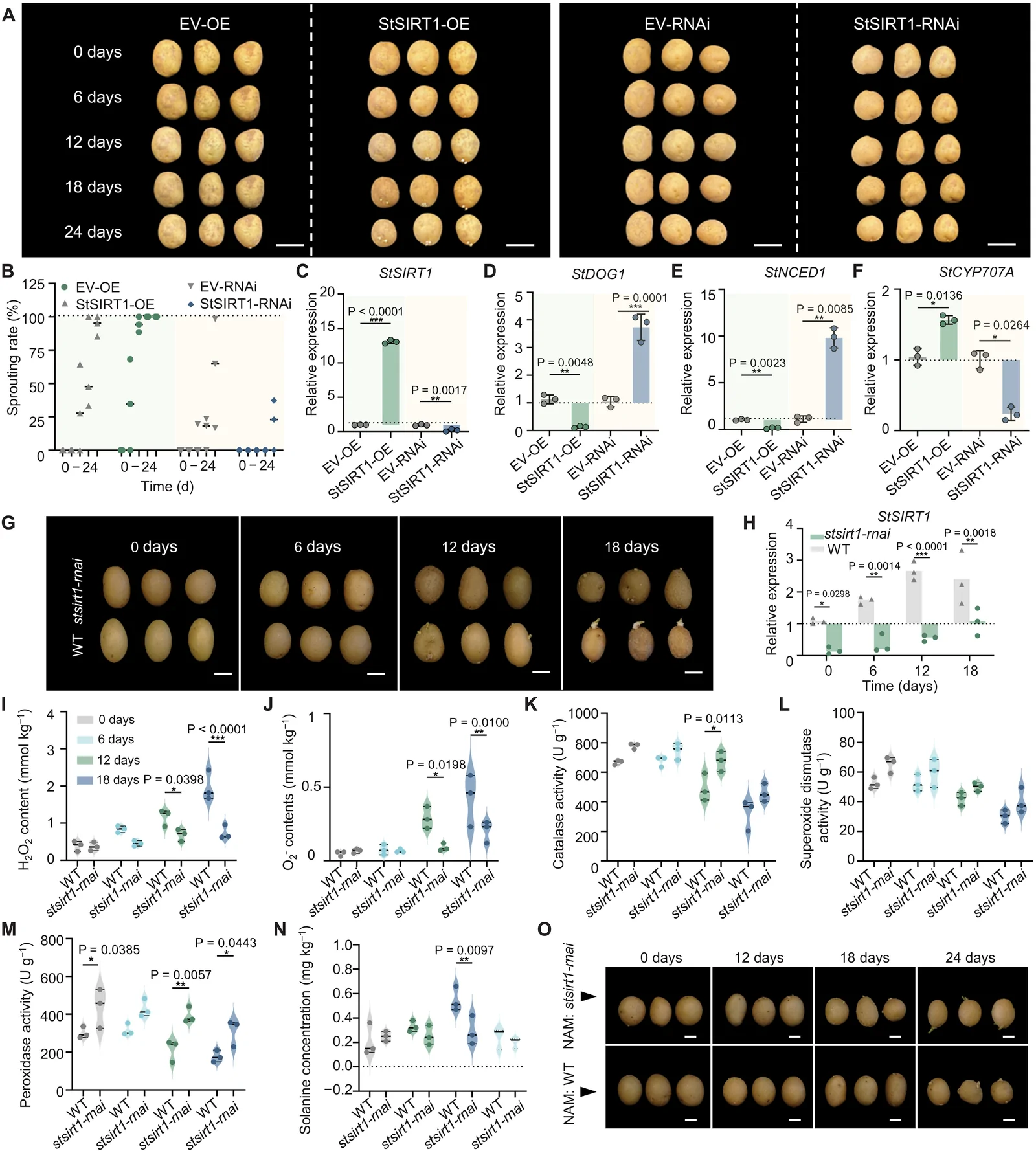
StSIRT1 negatively regulates potato dormancy. (**A**) Phenotypes of transiently transformed StSIRT1-OE and StSIRT1-RNAi potatoes. EV-OE and EV-RNAi lines served as controls. Potatoes were stored under dark conditions at 20°C for 24 days, with images recorded every 6 days. Scale bars, 5 cm. (**B**) Sprouting rate of StSIRT1-OE and StSIRT1-RNAi potatoes. (**C** to **F**) Expression levels of *StSIRT1*, *StDOG1*, *StNCED1*, and *StCYP707A* in StSIRT1-OE and StSIRT1-RNAi potatoes at 24 days; *EF-1*α was used as an internal control. (**G**) Phenotypes of stable transgenic *stsirt-rnai Atlantic* potatoes. Wild-type (WT) lines served as controls. Potatoes were stored under dark conditions at 20°C for 18 days, with images recorded every 6 days. Scale bars, 1 cm. (**H**) Expression levels of *StSIRT1* in *stsirt-rnai* potatoes; *EF-1*α was used as an internal control. (**I** and **J**) H_2_O_2_ and O_2_^–^ contents in *stsirt-rnai* and WT potatoes. (**K** to **M**) Catalase, superoxide dismutase, and peroxidase activities in *stsirt-rnai* and WT potatoes. (**N**) Solanine concentration in *stsirt-rnai* and WT potatoes. (**O**) Phenotypes of *stsirt-rnai* and WT potatoes in response to NAM (300 mM). Potatoes were stored under dark conditions at 20°C for 24 days, with images recorded every 6 days. Scale bars, 1 cm. Values are means ± SE; *n* = 3 for [(A) to (O)]. One-way ANOVA with Tukey’s test was used in [(C) to (F)]. Two-way ANOVA with Tukey’s multiple-comparison test was used in [(H) to (N)]. *P < 0.05, **P < 0.01, and ***P < 0.001.

To substantiate the role of StSIRT1 in dormancy regulation, we constructed stable *stsirt-rnai* transgenic potato lines in the “*Atlantic*” and “*Desiree*” cultivars (fig. S5A). Compared with *Desiree*, *stsirt-rnai* lines in *Atlantic* showed stronger sprouting inhibition from 12 days, suggesting that *Atlantic* better fits the dormancy-sprouting model (Fig. 2A and fig. S5B). Figure 2 (H and N) showed that *StSIRT1* expression was significantly higher in *stsirt-rnai* lines than in wild-type (WT) potatoes throughout the storage period, along with reduced solanine accumulation between 6 and 18 days. Previous studies showed that NAM promoted NADPH accumulation, reduced ROS levels, and thereby prolonged dormancy (*22*). To investigate the potential role of NAM in regulating StSIRT1-mediated redox homeostasis, we quantified ROS levels. O_2_^−^ and H_2_O_2_ levels increased during dormancy release in WT potatoes, whereas their accumulation was significantly reduced in *stsirt-rnai* lines from 12 days onward (Fig. 2, I and J). We further examined antioxidant enzyme activities involved in O_2_^−^ and H_2_O_2_ scavenging. Superoxide dismutase, catalase, and peroxidase activities were differentially regulated by StSIRT1 (Fig. 2, L and M). Notably, in *stsirt-rnai* lines, catalase activity peaked at 12 days during early dormancy release in response to a transient H_2_O_2_ burst, whereas peroxidase activity remained higher throughout storage, consistent with low but sustained H_2_O_2_ levels during dormancy. To verify the sensitivity of StSIRT1 to NAM, we treated *stsirt-rnai* lines with exogenous NAM. As shown in Fig. 2O, NAM reduced the sprouting phenotype in WT at 18 days, whereas it had no effect in *stsirt-rnai* lines. This difference became more evident at 24 days, indicating that StSIRT1 functions as a NAM-responsive deacetylase that negatively regulates dormancy maintenance. Considering the pivotal roles of NAM and *StSIRT1* in dormancy regulation, we sought to identify direct downstream targets of *StSIRT1* in the molecular cascade governing dormancy release.

### Dormancy is accompanied by global changes in histone acetylation levels

To determine whether lysine acetylation undergoes global remodeling in the transition from dormancy to sprouting, we profiled global lysine acetylation during potato storage and assessed NAM-dependent changes. Western blotting of bud eye tissue extracts confirmed extensive protein acetylation across a wide molecular weight range and revealed higher global acetylation levels in dormant tissues than in sprouting tissues (Fig. 3A). To characterize dormancy- or NAM-responsive acetylation events, we established natural physiological dormancy sprouting (NPDS) and NAM-treated dormancy sprouting (NTDS) groups and then conducted four-dimensional label-free acetyl-proteomic analysis (fig. S6A). Principal components and correlation analyses showed clear separation among groups with strong within-group reproducibility, indicating high data reliability (fig. S6, C and D). In total, 1041 acetylated proteins, 1872 acetylated peptides, and 1738 lysine acetylation sites were identified (Fig. 3B). The greatest overlap of acetylated proteins and sites occurred between 0 and 24 days in the NTDS group, indicating that NAM reduced acetylation changes associated with sprouting (Fig. 3D). Nearly half of the acetylated proteins contained a single modification site, most modified peptides were 7 to 20 residues in length, and more than 85% of proteins were quantified from at least two peptides, corroborating the data quality (Fig. 3E). Differential site analysis showed that acetylation loss increased as dormancy was released; NAM attenuated this deacetylation trend, as illustrated by volcano plots showing a reduced number of notably altered acetylation events in the NTDS group (Fig. 3C and fig. S6B). Our results indicate that NAM preserves protein acetylation during storage, thereby counteracting the deacetylation associated with sprouting. To comprehensively characterize protein acetylation during potato dormancy release, we identified acetylated histones and nonhistones. The acetylation atlas showed that the NPDS and NTDS groups contained only six evolutionarily conserved histones (containing 26 lysine acetylation sites), whereas 99.4% of the identified acetylated proteins were nonhistones, highlighting the broad regulatory role of nonhistone in potato dormancy (fig. S7) (*24*).

**Fig. 3.**
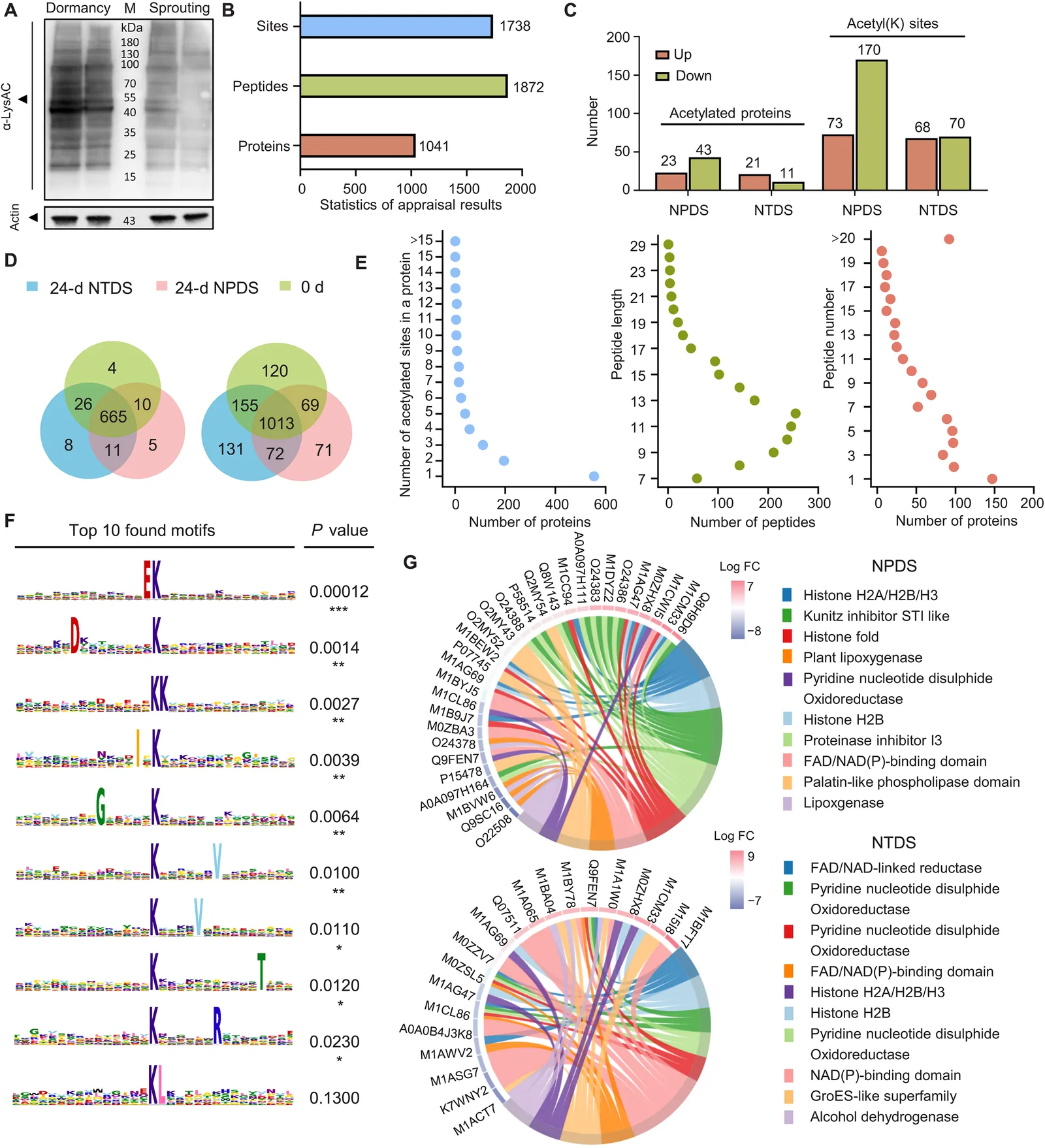
Global landscape of NAM-regulated acetylation in potato. (**A**) Immunoprecipitation analysis of total acetylated proteins in dormant and sprouting potato tissues. (**B**) Statistics of acetylated proteins, peptides, and acetylation sites identified by acetylome profiling. (**C**) Quantification of differentially acetylated sites and proteins between NTDS and NPDS groups. (**D**) Comparison of overlapping acetylated proteins within the total acetylome. Venn diagram illustrates the numbers of identified acetylated proteins and sites in the 0 days, 24-day NTDS, and 24-day NPDS groups. (**E**) Quality control analysis of acetylome data, including the number of acetylated lysine sites per protein, peptide length distribution, and peptide identification counts. (**F**) Top 10 most strongly enriched lysine acetylation motifs identified in the NTDS and NPDS groups. Each motif represents the sequence flanking the acetylated K, encompassing 10 amino acids on each side. (**G**) InterPro enrichment chord diagrams of differentially acetylated proteins; red indicates up-regulation, and blue indicates down-regulation. FC, fold change. *P < 0.05, **P < 0.01, and ***P < 0.001.

### Acetylome profiling identified StPRX3 as a NAM-responsive target of StSIRT1

To explore the features of potential targets and associated pathways, we performed enrichment analyses of motifs, InterPro domains, Gene Ontology (GO), and Kyoto Encyclopedia of Genes and Genomes (KEGG) annotations. Motif enrichment analysis identified EK^ac^ as the most enriched sequence, consistent with key acetylation patterns reported in rice seed dormancy and sprouting (Fig. 3F) (*25*). The conserved K^ac^K motif, also reported in wheat and maize, reflects a lysine-centered preference that facilitates recognition of acetylation sites by regulatory proteins (*15*). InterPro enrichment analysis identified conserved histone H2A, H2B, and H3 domains in both dormancy models, supporting a role for chromatin-based epigenetic regulation; enrichment of pyridine nucleotide–disulfide oxidoreductase and flavin adenine dinucleotide (FAD)/NAD(P)-binding domains indicated a central role for redox homeostasis in dormancy regulation (Fig. 3G). GO enrichment analysis comparing NPDS and NTDS groups identified enrichment of several conserved biological processes, including glycolysis, glucose metabolism, nucleosome assembly, and negative regulation of peptidase activity, along with molecular functions such as oxidoreductase activity and NAD(P) binding (fig. S8). Notably, NPDS showed enrichment of biosynthetic and plastid-related pathways, including fatty acid synthesis and chloroplast and amyloplast functions, consistent with metabolic reactivation during dormancy release. KEGG pathway analysis further implicated central carbon and amino acid metabolism, peroxisomal ROS scavenging, and solanine biosynthesis pathways, which are closely associated with sprout development (Fig. 4, A and B). Notably, NPDS was enriched for biosynthetic and plastid-related functions, whereas NTDS showed greater enrichment of antioxidant defense domains.

**Fig. 4.**
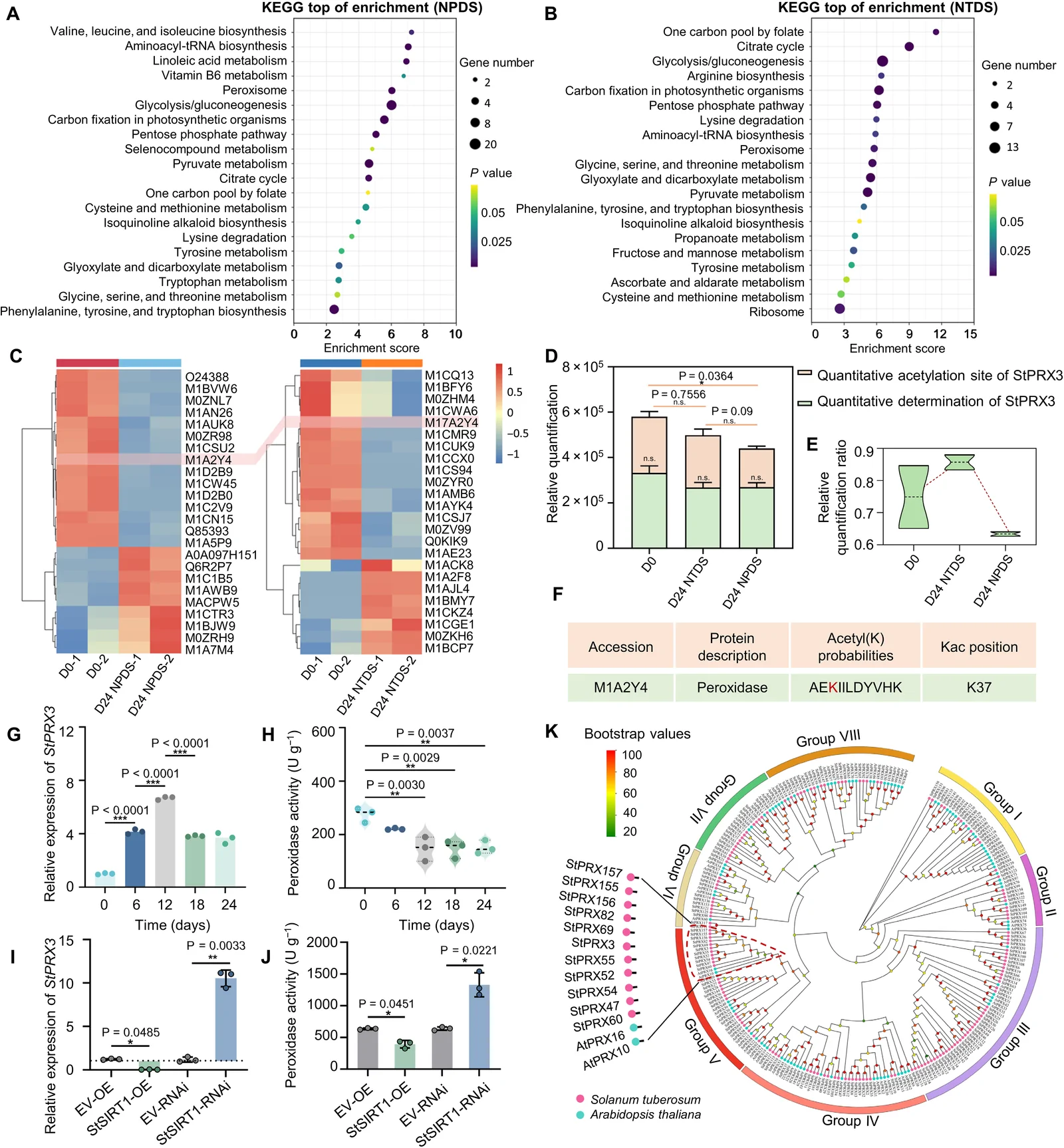
Identification of target proteins associated with potato dormancy. (**A** and **B**) KEGG enrichment analysis of lysine-acetylated proteins in NPDS and NTDS groups. Each circle denotes a KEGG term; circle size indicates the number of clustered acetylated proteins, and circle color indicates enrichment significance. tRNA, transfer RNA. (**C**) Clustered heatmap of the top 24 differentially acetylated proteins in NPDS and NTDS groups; red and blue represent increased and decreased abundance, respectively. M1A2Y4 is the sole overlapping protein between the two groups. (**D**) Protein and acetylation site abundance of M1A2Y4 (peroxidase, StPRX3) in NPDS and NTDS groups. D0, day 0; n.s., not significant. (**E**) Normalized relative quantification ratio, defined as the proportion of acetylated StPRX3 relative to total StPRX3 abundance. (**F**) Acetylation modification motifs and sites of M1A2Y4 identified by acetylome. (**G**) Expression levels of *StPRX3* during storage from 0 to 24 days; *EF-1*α was used as an internal control. (**H**) Dynamic of peroxidase activity during storage from 0 to 24 days. (**I**) Expression levels of *StPRX3* in StSIRT1-OE and StSIRT1-RNAi potatoes at 24 days; *EF-1*α was used as an internal control. (**J**) Dynamic of peroxidase activity in StSIRT1-OE and StSIRT1-RNAi potatoes at 24 days. (**K**) Phylogenetic analysis of the PRX family in *Solanum tuberosum* L. Values are means ± SE; *n* = 3 for [(G) to (J)]. One-way ANOVA with Tukey’s test was used in [(G) to (J)]. *P < 0.05, **P < 0.01, and ***P < 0.001.

To identify functional candidates from the acetylome data, we compared significantly differentially acetylated proteins between 0 and 24 days in both NPDS and NTDS groups. High-confidence acetylation sites were retained with localization probability ≥ 0.75 and delta score ≥ 8. Proteins with fold change ≥ 1.2 or ≤ 1/1.2 were considered significantly changed. We selected the top 24 differentially acetylated proteins from each cluster and identified their intersection between NPDS and NTDS. Only one protein, M1A2Y4 (peroxidase-like 3; StPRX3), was detected in both the NTDS and NPDS groups, showing consistent hyperacetylation during dormancy and strong deacetylation upon sprouting (Fig. 4C). Quantitative analysis further supported its relevance. At 24 days, StPRX3 acetylation decreased during dormancy release in the NPDS group, whereas no significant change was observed in the NTDS group, suggesting that NAM sustains StPRX3 acetylation (Fig. 4D). To correct for differences in protein abundance, we normalized StPRX3 acetylation intensity. Figure 4E showed that StPRX3 acetylation in the NTDS group was 1.45-fold higher than in the NPDS group at 24 days, while NPDS decreased to 0.85-fold of 0 days, reflecting that NAM treatment maintained elevated acetylation levels throughout storage (Fig. 4E). On the basis of the mass spectrometry data, we identified the peptide “AEKIILDYVHK” from StPRX3, in which the “EK” motif represents a potential acetylation site, indicating a regulatory acetylation hotspot at K37 (Fig. 4D). StPRX3 was categorized as a class III peroxidase (EC 1.11.1.7) with roles in H_2_O_2_ detoxification, lignification, and auxin metabolism, suggesting a role in potato dormancy via redox control (*26*). Expression analysis showed that *StPRX3* transcript levels initially increased by 6 days and subsequently declined at sprout onset, while its encoded peroxidase activity peaked during early storage and decreased to 50% by 24 days (Fig. 4, F and G). These findings indicate that *StPRX3* expression and peroxidase activity are closely associated with dormancy maintenance and that their decline coincides with oxidative accumulation during sprouting.

To clarify the regulatory relationship between *StSIRT1* and *StPRX3*, we examined *StPRX3* expression in StSIRT1-OE and StSIRT1-RNAi lines. *StPRX3* transcript levels were significantly lower in StSIRT1-OE potatoes, whereas levels in StSIRT1-RNAi potatoes were 112-fold higher than those in controls, indicating that StSIRT1 negatively regulates *StPRX3* expression (Fig. 4I). Consistent with these findings, peroxidase activity was 2.6-fold higher in StSIRT1-RNAi potatoes than in 35S:StSIRT1 lines (Fig. 4J).

### *StPRX3* positively regulates dormancy by modulating peroxidase activity

To elucidate the role of *StPRX3* in dormancy, we examined its sequence, structural features, and evolutionary conservation. *StPRX3* is located within a 1251-bp genomic fragment containing a 986-bp coding sequence that encodes a 327–amino-acid protein with a predicted molecular mass of 35.6 kDa (fig. S9A). To investigate the evolutionary relationships of StPRX3, we retrieved 158 StPRX proteins from the potato genome database (*Solanum tuberosum* v6.1). Phylogenetic analysis grouped these proteins into eight clusters (groups I to VIII), with StPRX3 assigned to group V and clustering with AtPRX55 and AtPRX65 (Fig. 4K). Bioinformatic analyses predicted that StPRX3 has a balanced hydropathicity profile, lacks transmembrane domains, and contains an N-terminal signal peptide (residues 1 to 22) (fig. S9, D to F). Promoter analysis of the 2-kb upstream region identified multiple cis-regulatory elements associated with auxin, jasmonate, low temperature, and light responsiveness, suggesting that StPRX3 expression is regulated by both hormonal and environmental cues (fig. S9B).

To investigate the role of StPRX3 in dormancy, we generated transient overexpression (StPRX3-OE) and RNAi (StPRX3-RNAi) constructs via Nimble Cloning and introduced them into tubers by vacuum infiltration. qRT-PCR analysis confirmed that *StPRX3* transcript levels in 35S: StPRX3 potatoes were 3.0-fold higher than those in EV controls, whereas levels in StPRX3-RNAi lines were reduced to 0.52-fold of control levels (Fig. 5B). Phenotypically, EV potatoes initiated sprouting by 18 days, whereas StPRX3-OE potatoes remained dormant for 24 days. In contrast, StPRX3-RNAi potatoes sprouted 12 days earlier than EV potatoes (Fig. 5A). At the molecular level, StPRX3 overexpression increased transcript levels of *StDOG1* and *StNCED1* by two- to fourfold and suppressed *StCYP707A1*, whereas *StCYP707A1* expression was 35-fold higher in StPRX3-RNAi potatoes than in controls (Fig. 5, C to E). These transcriptional changes were consistent with enzymatic alterations; peroxidase activity in StPRX3-OE tubers was 1.75-fold higher than in the EV-OE group, whereas PRX3-RNAi tubers showed approximately half of the control (Fig. 5F). StPRX3 also regulated H_2_O_2_ levels, with OE lines showing reduced H_2_O_2_ content and RNAi lines exhibiting an 87.5% increase relative to controls (Fig. 5G). In situ H_2_O_2_ staining confirmed these differences, revealing more intense staining in RNAi tissues (Fig. 5H). Moreover, expression of the NADPH oxidase *StRBOHA* was considerably elevated in StPRX3-RNAi potatoes (fig. S10, A and B). These results indicate that *StPRX3* modulates dormancy by maintaining peroxidase activity levels and constraining oxidative signaling.

**Fig. 5.**
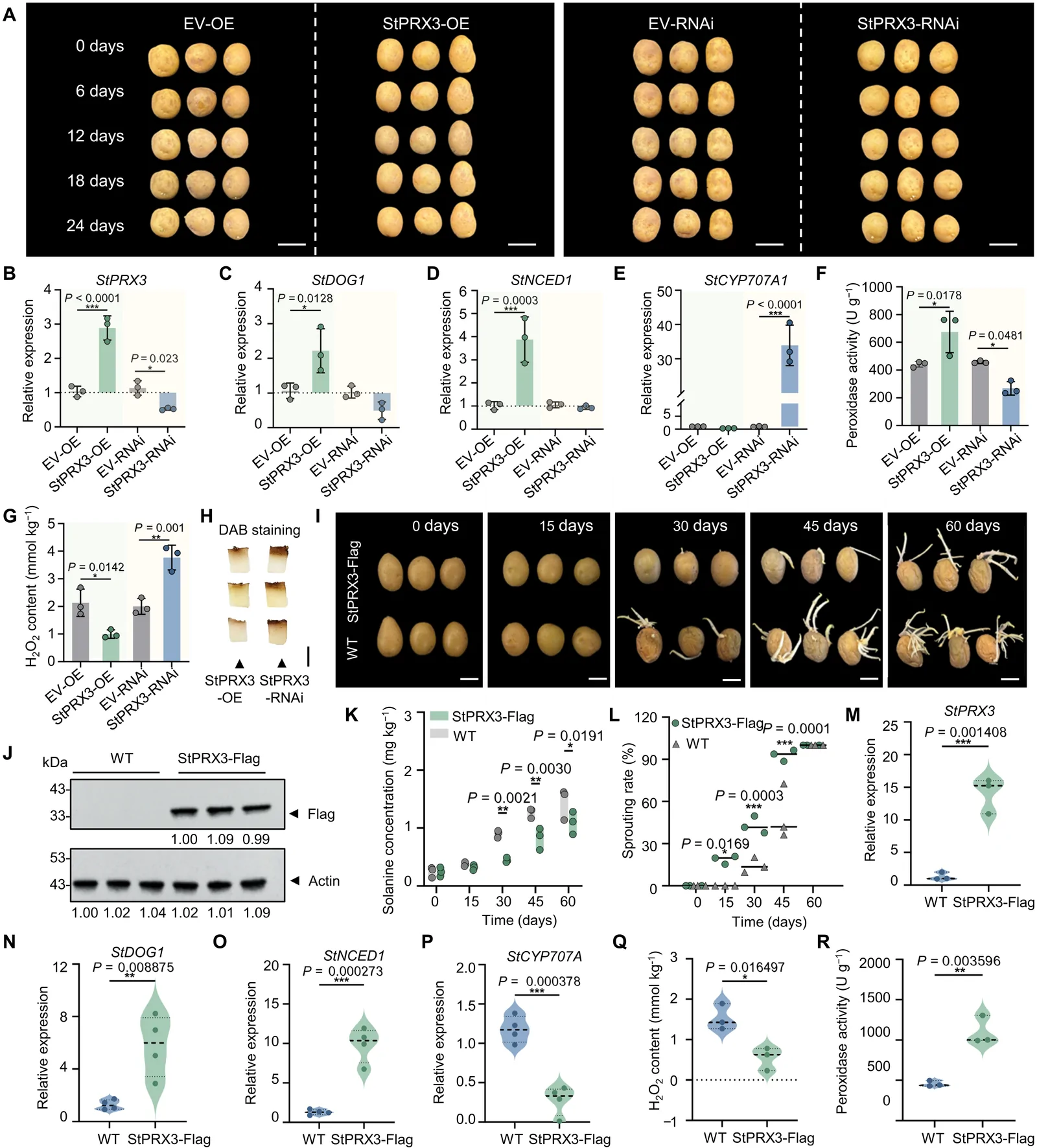
StPRX3 positively regulates potato dormancy. (**A**) Phenotypes of transiently transformed StPRX3-OE and StPRX3-RNAi potatoes. EV-OE and EV-RNAi lines served as controls. Potatoes were stored under dark conditions at 20°C for 24 days, with images recorded every 6 days. Scale bars, 5 cm. (**B** to **E**) Expression levels of *StPRX3*, *StDOG1*, *StNCED1*, and *StCYP707A* in StPRX3-OE and StPRX3-RNAi potatoes at 24 days; *EF-1*α was used as an internal control. (**F**) Peroxidase activity in StPRX3-OE and StPRX3-RNAi potatoes at 24 days. (**G**) H_2_O_2_ content in StPRX3-OE and StPRX3-RNAi potatoes at 24 days. (**H**) 3,3′-diaminobenzidine (DAB) staining of StPRX3-OE and StPRX3-RNAi potatoes at 24 days. Scale bar, 1 cm. (**I**) Phenotypes of stable transgenic StPRX3-Flag *Atlantic* potatoes. Potatoes were stored under dark conditions at 20°C for 60 days, with images recorded every 15 days. Scale bar, 1 cm. (**J**) Immunoblot analysis detecting the protein levels of StPRX3-Flag in stable transgenic potatoes. The protein levels were quantified using ImageJ. (**K**) Solanine concentration of StPRX3-Flag and WT potatoes. (**L**) Sprouting rate of StPRX3-Flag and WT potatoes. (**M** to **P**) Expression levels of *StPRX3*, *StDOG1*, *StNCED1*, and *StCYP707A* in StPRX3-Flag at 30 days; *EF-1*α was used as an internal control. (**Q**) H_2_O_2_ content in StPRX3-Flag at 30 days. (**R**) Peroxidase activity in StPRX3-Flag at 30 days. Values are means ± SE; *n* = 3 for [(A) to (M) and (Q) to (R)] and *n* = 4 for [(N) to (P)]. One-way ANOVA followed by Tukey’s test was used in [(B) to (G)]; two-way ANOVA with Tukey’s multiple-comparison test was used in [(K) and (L)]; Student’s *t* test (two-tailed) was used in [(M) to (R)]. *P < 0.05, **P < 0.01, and ***P < 0.001.

To substantiate the role of StPRX3 in dormancy control, we constructed stable *StPRX3-Flag* transgenic potatoes (*StPRX3-Flag*) (Fig. 5, I and J, and fig. S10B). Compared with WT plants, *StPRX3-Flag* seedlings exhibited reduced branching and increased height (fig. S10, D to F). Although peroxidase activity was significantly elevated, H_2_O_2_ levels were comparable to those in WT, reflecting limited oxidative stress during vegetative growth (fig. S10, G and H). At harvest, *StPRX3* expression was 23-fold higher in transgenic potatoes than in WT potatoes (Fig. 5M). During storage, with assessments conducted every 15 days, StPRX3-Flag tubers displayed greatly delayed sprouting between 15 and 30 days (Fig. 5I). By 60 days, both genotypes had fully sprouted; however, transgenic sprouts were shorter (Fig. 5I). Solanine content at 30 days was 50% lower in *StPRX3-Flag* than in WT tubers (Fig. 6K). The expression patterns of dormancy-associated genes further supported prolonged dormancy in *StPRX3-Flag* tubers; *StDOG1* and *StNCED1* were up-regulated, whereas *StCYP707A1* was suppressed (Fig. 5, N to P). *StPRX3-Flag* tubers accumulated less H_2_O_2_ than WT tubers and showed twofold higher peroxidase activity, supporting a model in which StPRX3 preserves dormancy by sustaining peroxidase-mediated H_2_O_2_ scavenging, whereas StPRX3 loss shifts the redox equilibrium toward ROS accumulation, thus accelerating sprouting (Fig. 5, Q and R).

**Fig. 6.**
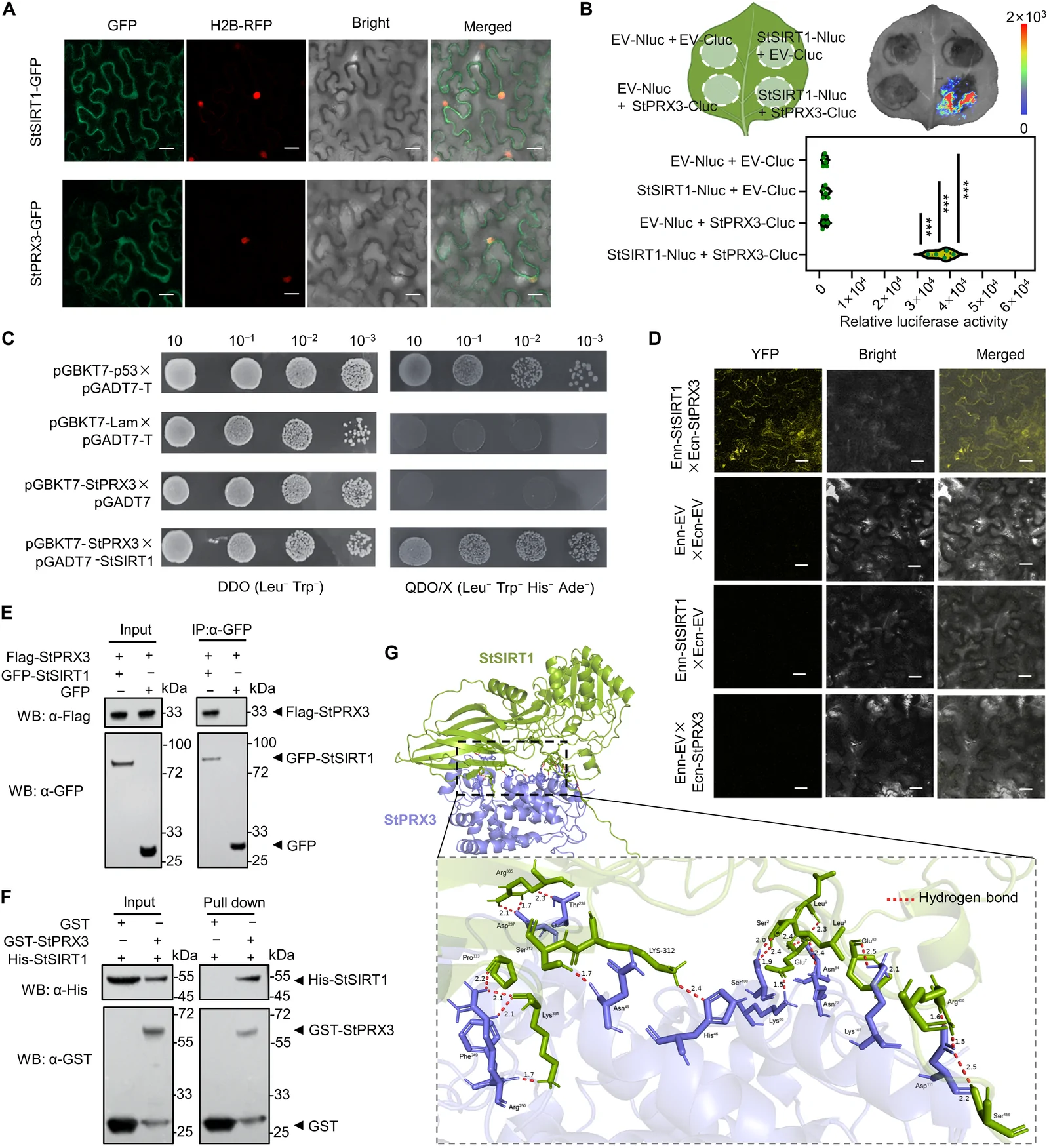
StSIRT1 interacts with StPRX3 in vitro and in vivo. (**A**) StSIRT1-GFP and StPRX3-GFP location in *N. benthamiana* H2B–red fluorescent protein (RFP) line. Scale bars, 20 μm. (**B**) LCA imaging showing the interaction between StSIRT1 and StPRX3 in *N. benthamiana* leaves; quantification of relative luminescence reflects protein-protein interaction levels. (**C**) Y2H assay showing interactions between pGADT7-StSIRT1 and pGBKT7-StPRX3 fusion proteins. Empty pGADT7 was used as the negative control. SD-L-T, SD/−Leu/−Trp medium; SD-L-T-H-A, SD/−Leu/−Trp/-His/−Ade medium. (**D**) BiFC assays showing interactions between StSIRT1 and Ecn-StPRX3 in the cytoplasm and nucleus. *N. benthamiana* leaves were cotransformed with StPRX3-cYFP or empty-cYFP and StSIRT1-nYFP or empty-nYFP. Scale bars, 20 μm. (**E**) Co-IP assay of StPRX3-Flag and StSIRT1-GFP fusion proteins in vivo. Empty GFP was used as the control. (**F**) GST pull-down assays showing interactions between His-StSIRT1 and GST-StPRX3 in vitro. GST alone was used as the control. (**G**) Molecular docking simulation showing predicted interactions between StSIRT1 and StPRX3. Red dotted lines indicate hydrogen bonding. Values are means ± SE; *n* = 5 for (B) and *n* = 3 for [(A) to (F)]. One-way ANOVA with Tukey’s test was used in (B). *P < 0.05, **P < 0.01, and ***P < 0.001.

To investigate temporal relationships among StSIRT1, StPRX3, peroxidase activity, and H_2_O_2_ accumulation, we integrated the time-course profiles into a heatmap analysis. As shown in fig. S11A, StSIRT1 remained low expression during 0 to 12 days, when StPRX3 expression and peroxidase activity increased, indicating enhanced H_2_O_2_ scavenging capacity. During 12 to 24 days, StSIRT1 expression increased, accompanied by reduced StPRX3 expression and peroxidase activity, weakening H_2_O_2_ scavenging capacity and promoting dormancy release. Under NAM treatment, this temporal transition was disrupted, with sustained StPRX3 expression and peroxidase activity, and reduced StSIRT1 induction, thereby reducing H_2_O_2_ accumulation from 6 days onward and delaying dormancy release. Correlation analysis revealed that StSIRT1 positively correlated with H_2_O_2_ accumulation and sprouting, whereas peroxidase activity showed negative correlations with these traits. StPRX3 positively correlated with peroxidase activity but negatively with H_2_O_2_, showing an StSIRT1-StPRX3-ROS regulatory axis during dormancy release (fig. S11B).

### K37 in StPRX3 is essential for its direct interaction with StSIRT1

Given the negative regulatory relationship between StSIRT1 and StPRX3, we performed subcellular localization analysis and found that both proteins were localized in the cytoplasm and nucleus, suggesting a potential functional association (Fig. 6A). We next tested whether StSIRT1 directly interacts with StPRX3. Luciferase complementation assays (LCAs) revealed strong luminescence upon coexpression of StSIRT1-Nluc and StPRX3-Cluc, whereas no signal was detected in control combinations, confirming a direct interaction in vivo (Fig. 6B). Yeast two-hybrid (Y2H) assays further demonstrated that yeast coexpressing StSIRT1-AD and StPRX3-BK grew robustly on selective medium, in contrast to negative controls, supporting this interaction (Fig. 6C). Bimolecular fluorescence complementation (BiFC) analysis showed that StSIRT1-StPRX3 complexes localized to both the cytoplasm and nucleus (Fig. 6D). Coimmunoprecipitation (Co-IP) assays provided additional in vivo validation because StPRX3-Flag was solely detected in StSIRT1–green fluorescent protein (GFP)–expressing samples (Fig. 6E). In vitro pull-down assays using purified StSIRT1-His and StPRX3–glutathione *S*-transferase (GST) verified a direct physical association, whereas the GST control showed no binding (Fig. 6F). To gain structural insight into the interaction interface, molecular docking analysis using the HDOCK server predicted a stable StSIRT1-StPRX3 complex with a HADDOCK score of −32 ± 8.1 and a root mean square deviation of <4.3 ± 0.1 Å (Fig. 6G). The predicted buried surface area of 3113 ± 267 Å^2^ indicates an extensive interaction interface, and the negative *z*-score (−1.0) supports model reliability. Energy decomposition analysis revealed dominant contributions from van der Waals interactions (−69.3 ± 12.0 kcal mol^−1^) and electrostatic forces (−445.9 ± 75 kcal mol^−1^), suggesting that electrostatic attraction plays a key stabilizing role in StSIRT1-StPRX3 complex formation.

To identify potential acetylation sites in StPRX3, we performed DeepPLA-based prediction and identified 20 candidate sites (fig. S13). Integrating these results with identified acetylation sites from the acetylome, we propose that K37 is a key acetylation site in StPRX3 (Fig. 4D). Deacetylase substrate interaction prediction further showed that SIRT1 preferentially targets K37 with high probability (51.68%), suggesting a potential functional association between StSIRT1 and StPRX3 (Fig. 7A). AlphaFold3 modeling and PyMOL visualization generated the acetylated StPRX3 structure. Figure 7B shows that K37 acetylation disrupts electrostatic interaction with Asp^41^ (D41) and induces local conformational changes in StPRX3, which may affect enzymatic activity. Molecular docking revealed a twofold reduction in binding affinity between StSIRT1 and acetylated StPRX3 compared with the unmodified form, suggesting that K37 deacetylation facilitates the interaction between StSIRT1 and StPRX3 (Fig. 7C). Structural domain analysis shows that K37 is located at the entry point of the plant peroxidase-like superfamily domain and lies near residues forming the catalytic pocket, suggesting that acetylation at this position alters enzyme conformation or activity (Fig. 7C). To test this hypothesis, we substituted K37 with glutamine (K37Q) to mimic the acetylated state and with arginine (K37R) to mimic the deacetylated state; both substitutions were confirmed by sequencing (fig. S14A). Y2H assays and LCAs showed that substitution of K37 with either Q or R abolished StSIRT1-StPRX3 binding, indicating that K37 is a critical contact residue recognized by StSIRT1 and that its acetylation functions as a molecular switch through which StSIRT1 directly modulates StPRX3 function (Fig. 7, E and F).

**Fig. 7.**
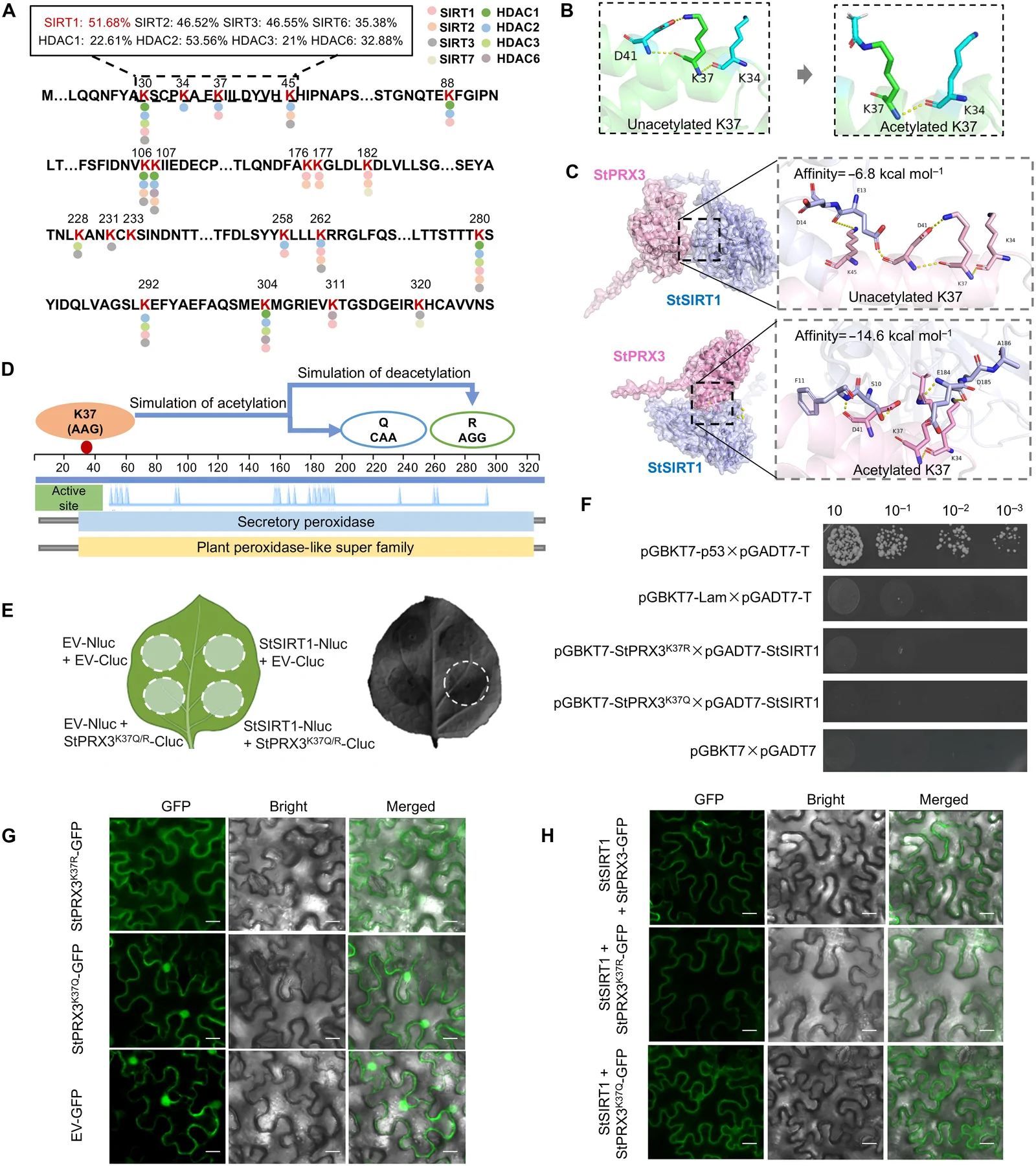
K37 acetylation serves as a switch for StSIRT1 to directly modulate StPRX3. (**A**) Predicted acetylation sites of StPRX3 and the corresponding deacetylases associated with each site. The boxed region indicates the binding probability of deacetylases at the K37. (**B**) Local conformational changes induced by K37 acetylation. D41 indicates the aspartic acid residue at position 41, and K34 represents the lysine residue at position 34. (**C**) Molecular docking analysis of StSIRT1 with StPRX3 acetylated at K37. (**D**) Schematic illustration of functional domains in StPRX3. The relative position of the differentially acetylated lysine site (K37) is indicated. K37 was substituted with glutamine (K37Q) to mimic acetylation and with arginine (K37R) to mimic the deacetylated state. (**E**) LCA imaging showing the interaction between StSIRT1-Nluc and StPRX3^K37R^-Cluc or StPRX3^K37Q^-Cluc in *N. benthamiana* leaves; quantification of relative luminescence reflects protein-protein interaction levels. (**F**) Y2H assay showing interactions between pGADT7-StSIRT1 and pGBKT7-StPRX3^K37R^ or pGBKT7-StPRX3^K37Q^ fusion proteins. Empty pGADT7 was used as the negative control. (**G**) Subcellular localization showing that StPRX3^K37R^ is predominantly localized in the cytoplasm, whereas StPRX3^K37Q^ is localized in both the cytoplasm and nucleus. *N. benthamiana* leaves expressing enhanced GFP (eGFP) were used as the negative control. Scale bars, 20 μm. (**H**) Subcellular localization of transiently coexpressed eGFP-StPRX3^K37R/Q^ and Flag-StSIRT1. Coexpression of eGFP-StPRX3 and Flag-StSIRT1 was used as the negative control. Scale bars, 20 μm. *n* = 3 for [(E) to (H)].

### Acetylation of StPRX3^K37^ enhances peroxidase activity and promotes nuclear localization

To explore whether K37 acetylation modulates *StPRX3* function, we purified GST-tagged *StPRX3* and its acetylation-mimic (*StPRX3^K37Q^*; K37→glutamine, K37Q; Q denotes glutamine) and deacetylation-mimic (*StPRX3^K37R^*) variants (fig. S14). Western blotting with an anti–acetyl-lysine antibody showed substantially lower acetylation in both *StPRX3*^K37Q^ and *StPRX3*^K37R^ compared with *StPRX3*, whereas *StPRX3*^K37Q^ retained acetylation levels comparable to those *of StPRX3*^K37R^, indicating that K37 is the key acetylation site among multiple predicted sites in StPRX3 (Fig. 8A). Although mutation at K37 affects antibody recognition of the epitope and weakens acetylation signal of StPRX3, the amino acid substitution introduces charge and structural changes that may affect enzymatic activity. Consistent with this, enzymatic assays showed that K37 acetylation is associated with higher catalytic activity. The deacetylation-mimic *StPRX3*^K37R^ exhibited a significant reduction in peroxidase activity relative to the activities of the acetyl-mimic *StPRX3*^K37Q^, whereas *StPRX3*^K37Q^ retained activity comparable to *StPRX3* (Fig. 8B). These results suggest that acetylation at StPRX3^K37^ stabilizes an active conformational state, whereas loss of this modification shifts StPRX3 toward a less active configuration. Subcellular localization analysis revealed that the K37Q mutation enhanced nuclear localization, whereas *StPRX3^K37R^* and StPRX3 displayed similar distribution patterns, indicating that mutation at K37 alters charge-dependent properties, which may influence the intracellular trafficking or retention of StPRX3 (Fig. 7D). Coexpression of StPRX3 with *StSIRT1-Flag* reduced nuclear localization of both StPRX3 and StPRX3^K37Q^ but did not affect localization of *StPRX3*^K37R^, indicating that acetylation at K37 enhances the nuclear localization signal of StPRX3, whereas StSIRT1-dependent deacetylation attenuates K37-driven nuclear targeting (Fig. 7E). These findings demonstrate that K37 acetylation of *StPRX3* enhances its peroxidase activity and promotes nuclear localization, whereas deacetylation by *StSIRT1* diminishes both effects.

**Fig. 8.**
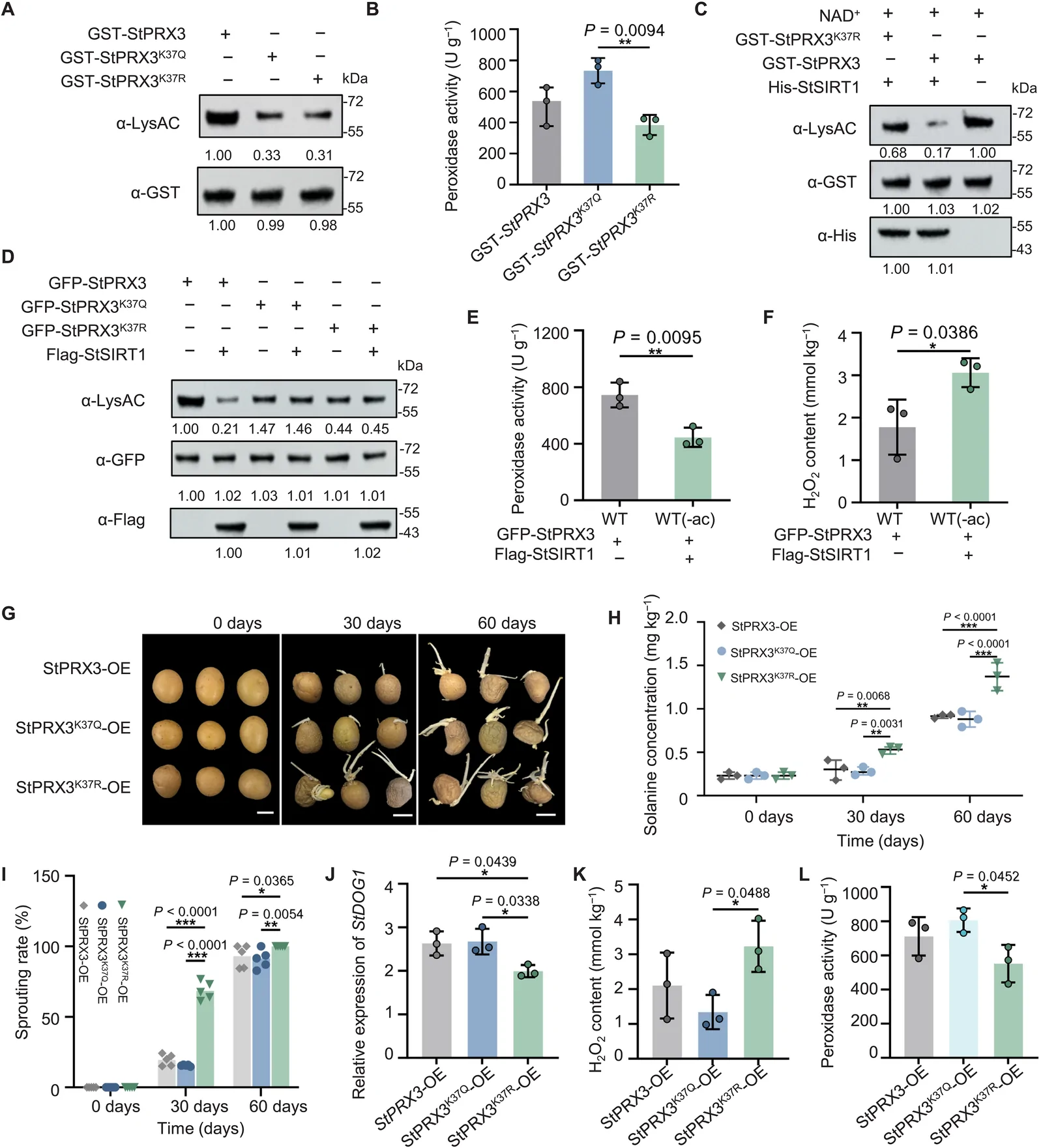
StSIRT1-mediated K37 deacetylation modulates StPRX3 function. (**A**) Acetylation levels of purified GST-StPRX3 and GST-StPRX3^K37R/Q^; the protein levels were quantified using ImageJ. (**B**) Peroxidase activity of purified GST-StPRX3 and GST-StPRX3^K37R/Q^. (**C**) Purified His-StSIRT1 deacetylated GST-StPRX3 and GST-StPRX3^K37R^ in vitro; the protein levels were quantified using ImageJ. (**D**) Flag-StSIRT1 deacetylates GFP-StPRX3 and GFP-StPRX3^K37R/Q^ in vivo; the protein levels were quantified using ImageJ. (**E**) Peroxidase activity in *N. benthamiana* leaves coexpressing GFP-StPRX3 and Flag-StSIRT1. (**F**) H_2_O_2_ levels in *N. benthamiana* leaves coexpressing GFP-StPRX3 and Flag-StSIRT1. (**G**) Phenotypes of stable transgenic StPRX3-OE, StPRX3^K37Q^-OE, and StPRX3^K37R^-OE *Atlantic* potatoes. Potatoes were stored under dark conditions at 20°C for 60 days, with images recorded every 30 days. Scale bars, 1 cm. (**H**) Solanine concentrations in StPRX3-OE, StPRX3^K37Q^-OE, and StPRX3^K37R^-OE transgenic potatoes. (**I**) Sprouting rates of StPRX3-OE, StPRX3^K37Q^-OE, and StPRX3^K37R^-OE transgenic potatoes. (**J**) Expression levels of *StDOG1* in StPRX3-OE, StPRX3^K37Q^-OE, and StPRX3^K37R^-OE transgenic potatoes; *EF-1*α was used as an internal control. (**K**) H_2_O_2_ content in StPRX3-OE, StPRX3^K37Q^-OE, and StPRX3^K37R^-OE transgenic potatoes at 60 days. (**L**) Peroxidase activity in StPRX3-OE, StPRX3^K37Q^-OE, and StPRX3^K37R^-OE transgenic potatoes at 60 days. Values are means ± SE; *n* = 3 for [(A) to (L)]. One-way ANOVA with Tukey’s test was used in (B) and [(J) to (L)]; Student’s *t* test (two-tailed) was used in [(E) and (F)]; two-way ANOVA with Tukey’s multiple-comparison test was used in (I). *P < 0.05, **P < 0.01, and ***P < 0.001.

### StSIRT1-mediated deacetylation of StPRX3^K37^ suppresses peroxidase activity and promotes dormancy release

Next, we assessed whether *StSIRT1* catalyzes deacetylation of *StPRX3* at K37 using complementary in vitro and in vivo assays. Incubation of recombinant GST-StPRX3 with His-StSIRT1 in the presence of NAD^+^ reduced the acetyl-lysine signal by 83%, whereas the deacetylation-mimic GST-StPRX3^K37R^ retained higher acetylation under identical conditions, indicating that StSIRT1 directly deacetylates StPRX3 in vitro, while the K37R mutation abolishes this effect, confirming K37 as the principal StSIRT1-sensitive residue (Fig. 8C and fig. S15). Similar results were obtained in vivo. In in vivo assays, acetylation of GFP-StPRX3^K37R^ and GFP-StPRX3^K37Q^ was lower than that of GFP-StPRX3; coexpression of Flag-StSIRT1 reduced acetylation of GFP-StPRX3 by 79% but did not significantly affect acetylation of GFP-StPRX3^K37Q^ or GFP-StPRX3^K37R^ (Fig. 8D and fig. S15). Functional analyses showed that *StSIRT1*-mediated deacetylation decreased peroxidase activity by 12.33% and increased H_2_O_2_ accumulation by 17.58% in agroinfiltrated tobacco leaves, indicating that deacetylation compromises the H_2_O_2_-scavenging capacity of *StPRX3* and disrupts redox balance (Fig. 8, E and F). To verify the role of K37 acetylation in planta, we generated *StPRX3^K37R^* and *StPRX3^K37Q^* transgenic potato lines. During storage, *StPRX3^K37R^* potatoes exhibited earlier dormancy release and more pronounced shrinkage compared with *StPRX3* and *StPRX3^K37Q^* lines (Fig. 8, G to I). Accordingly, solanine content was substantially higher in *StPRX3^K37R^* potatoes than in *StPRX3* and *StPRX3^K37Q^* lines, which showed no significant difference (Fig. 8H). Expression of *StDOG1* was lowest in the *StPRX3^K37Q^* line, suggesting a positive association between K37 acetylation and dormancy maintenance (Fig. 8J). Moreover, *StPRX3^K37Q^* potatoes exhibited 30% higher peroxidase activity and significantly lower H_2_O_2_ content compared with *StPRX3^K37R^* potatoes, confirming that *StSIRT1*-driven deacetylation of *StPRX3^K37^* represses peroxidase activity and perturbs redox balance (Fig. 8, K and L).

On the basis of the present findings, we propose a regulatory model in which NAM modulates potato dormancy through an StSIRT1-StPRX3-H_2_O_2_ signaling cascade (Fig. 9). During natural dormancy release, *StSIRT1* expression is strongly up-regulated, facilitating direct interaction with *StPRX3* and deacetylation at K37. This deacetylation attenuates peroxidase activity, thereby reducing H_2_O_2_ scavenging capacity, promoting ROS accumulation in eye tissues, and shifting cellular redox balance toward an oxidative state that triggers sprouting. Exogenous NAM inhibits *StSIRT1* activity, thus maintaining *StPRX3* acetylation and enhancing its H_2_O_2_-scavenging capacity, reestablishing redox homeostasis, and prolonging dormancy.

**Fig. 9.**
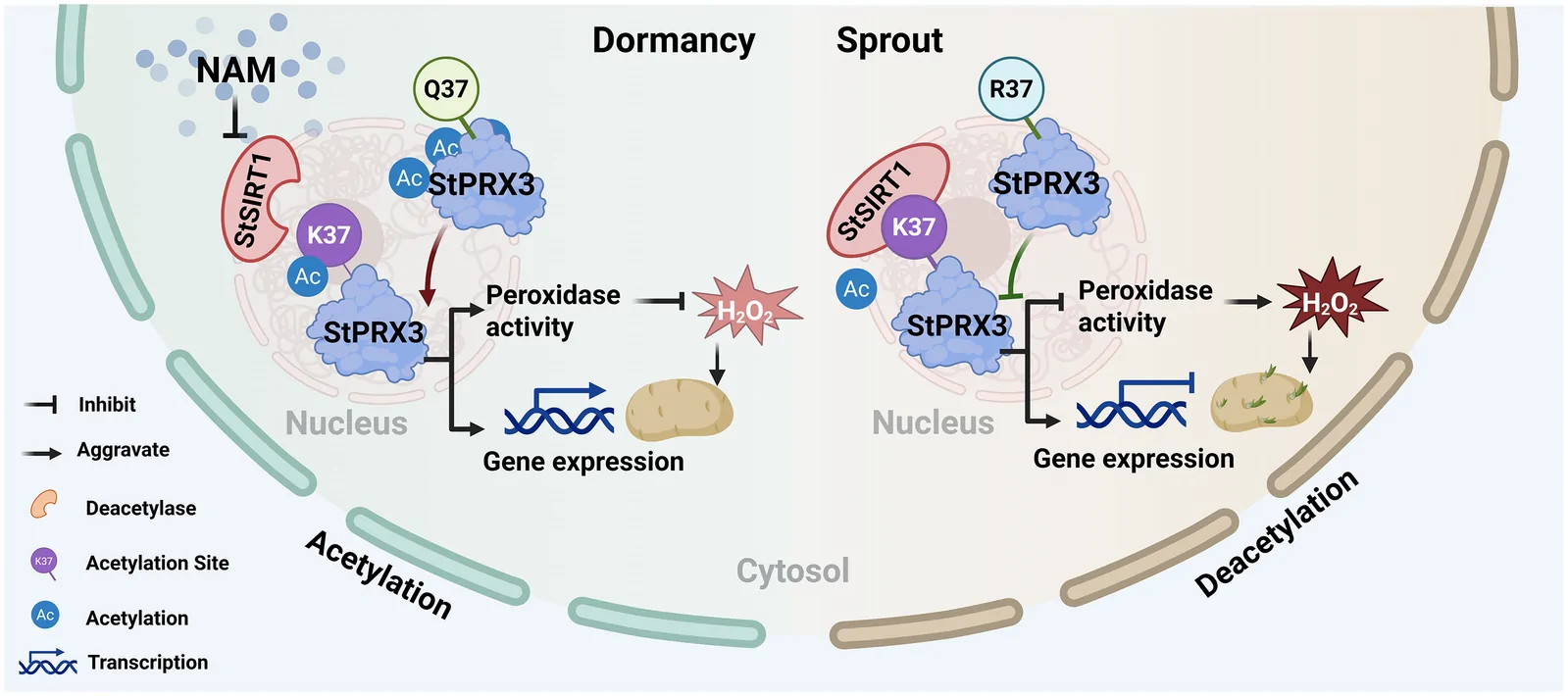
Proposed model for regulation of potato dormancy by the StSIRT1-StPRX3-H_2_O_2_ cascade. During dormancy release, StSIRT1 levels increase, and StPRX3 is recruited, leading to reduced StPRX3^K37ac^ levels. Consequently, peroxidase activity is suppressed, H_2_O_2_-scavenging capacity is diminished, and dormancy release is triggered. NAM inhibits *StSIRT1* activity, thus maintaining *StPRX3*^K37^ acetylation, restoring redox homeostasis, and prolonging potato dormancy. The figure was created in BioRender. Li, L. (2026) https://BioRender.com/4mp2r2v, and was licensed under CC BY 4.0.

## DISCUSSION

NAM, the bioactive amide form of niacin, is widely used as a dietary supplement to prevent pellagra by alleviating niacin deficiency–related symptoms, such as dermatitis and skin flushing (*27*–*29*). Because of its small molecular size and ability to cross the blood-brain barrier, NAM exerts neuroprotective effects in neurodegenerative disorders and is used as a chemopreventive and therapeutic agent for autoimmune and dermatological diseases (*23*, *27*, *30*, *31*). In plants, NAM contributes to cellular redox homeostasis by maintaining the NAD(P)/NAD(P)H balance, thereby protecting cells from oxidative stress–induced damage (*22*, *32*). As a precursor of NAD^+^, NAM inhibits SIRT deacetylase activity by competitively occupying the NAD^+^-binding pocket, thus modulating the acetylation status of key regulatory proteins (*33*). First identified as silent information regulators, SIRTs are conserved lysine deacetylases involved in a broad range of biological processes, including energy metabolism, oxidative stress responses, DNA repair, and regulation of cell death (*34*, *35*). Recent studies have revealed their diverse roles in plant growth and stress adaptation. In grape, VvSIRT2 is associated with photosynthetic regulation (*33*); in tomato, FolSIRT2 deacetylates the serine/arginine kinase FolGSK3 at K271 to enhance enzymatic activity and promote fungal pathogenicity (*36*); in rice, OsSIRT1 suppresses glyceraldehyde-3-phosphate dehydrogenase activity through deacetylation, thus modulating carbon metabolism during seedling development (*34*, *37*, *38*); and in *Arabidopsis*, AtSRT1 enhances stability of the transcriptional repressor AtMBP-1 via deacetylation, whereas AtSIRT2 contributes to seed dormancy and abiotic stress responses through H4K8 deacetylation, repressing *VAMP714* transcription and promoting germination under salt stress (*39*). Despite these advances, the physiological roles of NAM-mediated deacetylase inhibition in plants remain poorly understood. Our previous work demonstrated that exogenous NAM notably prolongs postharvest potato dormancy while preserving tuber quality, suggesting that NAM may regulate dormancy transitions through interference with SIRT-dependent deacetylation pathways.

Potato dormancy includes endodormancy, paradormancy, and ecodormancy. During endodormancy, internal factors suppress dormancy release potential, preventing sprouting even under favorable conditions. This is followed by a second phase (paradormancy and ecodormancy), in which bud meristems gradually regain responsiveness to physiological and environmental cues, including H_2_O_2_ levels, temperature, and humidity (*40*). Expression profiling showed that StSIRT1 exhibited low expression during dormancy release, consistent with a limited role at endodormancy. Its expression increases during the later stage of dormancy and associated with H_2_O_2_ accumulation, defining its functional window (Fig. 1G and fig. S1, B and C). This pattern indicates that StSIRT1 mainly functioned during paradormancy and ecodormancy. Through functional validation, we identified StSIRT1 as a NAM-responsive deacetylase that negatively regulates dormancy by limiting H_2_O_2_ scavenging (Fig. 2).

Acetylation undergoes dynamic regulation during plant dormancy and sprouting (*41*–*44*). In poplar, acetylation of H2A and H2B substantially increases during bud break, whereas in alfalfa seeds, lysine acetylation peaks 24 hours after imbibition; coordinated action of HDA19 and SUVH5 on H3 acetylation at the *ABI3* and *RGA* loci regulates seed sprouting (*18*, *19*, *41*). Consistent with these observations, the present study showed that global protein acetylation levels were significantly higher in dormant potato tissues than in sprouting tissues, suggesting that acetylation functions to suppress premature metabolic activation (Fig. 3A). Previous studies have revealed that bromoethane-induced dormancy release coincides with transient increases in H4 and H3.1/3.2 acetylation, indicating that epigenetic events accompany tuber sprouting (*45*). Acetyl-proteomic analyses across diverse plant species, including pea, rice, alfalfa, wheat, spruce, maize, morning glory, cassava, tobacco, strawberry, tea, and poplar, have demonstrated that nonhistone acetylation is widespread and implicated in developmental and metabolic processes (*15*, *16*, *18*, *20*, *24*, *25*, *46*–*51*). Although regulatory roles of nonhistone acetylation in plants are increasingly recognized, its involvement in dormancy and sprouting remains relatively understudied. Our acetyl-proteomic profiling revealed that 99.4% of acetylated proteins in potato were nonhistones, emphasizing a central role for nonhistone acetylation in potato dormancy regulation. Comparative analysis showed that NAM treatment attenuated the deacetylation associated with natural sprouting, suggesting that NAM preserves acetylation patterns to delay dormancy release (Fig. 3, B to D). Plant acetylated proteins are enriched in multiple metabolic pathways and regulate metabolic processes via modulation of protein function. The NPDS group exhibited enrichment of biosynthetic pathways, including fatty acid biosynthesis, chloroplast and plastid function, and amino acid metabolism (e.g., valine, leucine, and isoleucine biosynthesis), indicating that dormancy release activates cellular reprogramming and sprout development (fig. S8, A and B). InterPro enrichment analysis further identified histone H2A/H2B/H3, pyridine nucleotide–disulfide oxidoreductase, and FAD/NAD(P)-binding domains in both natural and NAM-treated models, suggesting that dormancy release involves coordinated chromatin-based epigenetic reprogramming and redox-associated protein acetylation, which together contribute to the maintenance of tuber redox homeostasis (Fig. 3G).

Dormancy release in potato is strongly associated with the generation of H_2_O_2_, a pivotal signaling molecule involved in meristem reactivation and cell wall loosening during sprouting (*7*, *11*, *52*). The precise mechanisms that regulate H_2_O_2_ levels during dormancy transition remain poorly understood. Among NAM-responsive proteins identified in the acetylome, the class III peroxidase StPRX3 emerged as a key redox regulator of dormancy (Fig. 4, C to E). Our results showed that the increase in H_2_O_2_ during sprouting was closely associated with StPRX3 deacetylation and reduced peroxidase activity, whereas catalase and superoxide dismutase activities showed no consistent changes (Fig. 4, G and H, and fig. S1, D to F). Peroxidases function as redox sensors in plants, maintain cellular redox homeostasis by scavenging H_2_O_2_ and other peroxides, and participate in diverse physiological processes, including growth and development, stress protection, and circadian regulation (*53*–*55*). Peroxidases are canonical dormancy markers in several plant species, such as barley, *Arabidopsis*, and buckwheat (*56*–*60*). Under stress conditions, peroxidase overexpression prolonged dormancy, which was associated with protection of seed nuclear DNA from oxidative damage (*61*, *62*). A recent study revealed a dual role for peroxidases in dormancy regulation, depending on involvement in cell wall metabolism (*63*). Seed-sprouting rates of *Arabidopsis* PRX2-PRX25 double mutants and PRX2-PRX25-PRX71 triple mutants were lower than those of WT plants (*64*). AtPRX16, AtPRX62, AtPRX69, and AtPRX71 strengthened cell walls through lignin polymerization, thereby counterbalancing hypocotyl growth stress and preventing premature seed sprouting (*63*). In contrast, AtPRX04 was expressed during dormancy release and facilitated cell wall loosening via nonenzymatic polysaccharide cleavage, creating space for bud growth (*63*). Functional specificity of peroxidases is closely linked to subcellular localization (*55*). Chloroplastic PRX1 and PRXQ are associated with energy metabolism; cytosolic PRX-B-D is linked to defense responses; pollen-specific PRXII-C and PRXII-D contribute to protection against desiccation-induced oxidative stress; and nuclear-localized peroxidases have been implicated in seed dormancy maintenance, indicating that localization confers distinct physiological roles (*65*–*67*). Although roles of peroxidases in dormancy have been identified in model plants, their functions in potato dormancy remain unclear. In potato, StPRX3 was detected in both the cytoplasm and nucleus, indicating potential involvement in coordinating redox regulation across cellular compartments (*56*). We found that *StPRX3* expression and peroxidase activity peaked during deep dormancy and declined as sprouting initiated, coincident with increased H_2_O_2_ accumulation (Fig. 4, G and H). In addition, StPRX3 expression showed a negative correlation with both solanine content and StSIRT1 expression (fig. S11B). Functional analyses demonstrated that transient or stable overexpression of StPRX3 enhanced peroxidase activity, reduced H_2_O_2_ accumulation, and delayed sprouting, whereas StPRX3 silencing resulted in premature ROS accumulation and accelerated dormancy release (Fig. 5). Collectively, these results identify StPRX3 as a redox-dependent modulator of potato dormancy.

Peroxidase-specific lysine acetylation is strongly associated with maintenance of peroxidase activity (*68*). Although acetyl-proteomic studies have shown that peroxidase-specific acetylation is linked to seed sprouting in rice, the underlying functional and regulatory mechanisms remain largely unknown (*69*). Our proteomic analysis revealed that StPRX3 acetylation is positively associated with dormancy maintenance in potato, indicating that StPRX3 acetylation is a critical regulator of peroxidase activity (Fig. 4, E and F). Identification of the enzyme responsible for modulating StPRX3 acetylation is essential to elucidate its regulatory mechanism. Given that NAM inhibits SIRT-type deacetylases, we hypothesized that StSIRT1 regulates StPRX3 acetylation. Increased StSIRT1 abundance and solanine accumulation during dormancy release nearly coincided with reduced *StPRX3* transcript levels and decreased peroxidase activity (fig. S11A). Subcellular localization analyses showed that both StSIRT1 and StPRX3 were present in the cytoplasm and nucleus, indicating potential functional interaction within shared cellular compartments (Fig. 6A). Functional assays further demonstrated that StSIRT1 negatively regulates *StPRX3* expression and peroxidase activity, resulting in increased H_2_O_2_ accumulation (Fig. 4, I and J). Multiple in vitro and in vivo approaches confirmed a direct physical interaction between StSIRT1 and StPRX3 (Fig. 6, B to F). Molecular docking simulations and structural visualization using HDOCK generated reliable models, revealing that electrostatic interactions constitute the primary driving force of the interaction; additional stabilization is provided by van der Waals forces (Fig. 6G).

Lysine acetylation at specific catalytic or structural residues exerts a profound influence on enzyme conformation and activity. Previous studies have shown that SIRT deacetylases dynamically regulate antioxidant enzyme activity. For instance, elevated ROS levels activate *SIRT3* transcription, which increases superoxide dismutase 2 deacetylation and enhances ROS scavenging (*70*); in *Ganoderma lucidum*, GLSIRT1-mediated deacetylation of GLCAT suppresses catalase activity, leading to intracellular ROS accumulation and altered secondary metabolism (*71*). In the present study, acetylome-based mass spectrometry, combined with molecular docking and computational prediction, identified K37 of StPRX3 as a potential key acetylation site (Fig. 7, A to C). The site-directed mutagenesis of K37 in StPRX3 demonstrated its critical role in enzyme regulation; mimicking K37 acetylation enhanced peroxidase activity, whereas mimicking deacetylation greatly reduced activity (Figs. 7D and 8, A and B). Acetylation at K37 also enhanced nuclear localization, indicating that this modification influences catalytic efficiency and spatial signaling functions (Fig. 7G). Substitution of K37 with R or Q abolished binding to StSIRT1, suggesting that this residue is essential for StSIRT1-mediated deacetylation (Fig. 7, E and F). Colocalization analyses further showed that StSIRT1-dependent deacetylation reduced nuclear localization of StPRX3, implying that acetylation-dependent subcellular partitioning contributes to the regulation of redox signaling (Fig. 7H). Although the K37 mutation abolished StSIRT1-mediated deacetylation of StPRX3^K37Q/R^, the charge alteration introduced by the substitution affected enzymatic activity, with StPRX3^K37Q^ exhibiting higher peroxidase activity than StPRX3^K37R^ (Fig. 8, B to D). In vitro and in vivo deacetylation assays confirmed that StSIRT1 directly removed acetyl groups from StPRX3 at K37, suppressing enzymatic activity. This inhibition reduced H_2_O_2_-scavenging capacity, disrupted redox equilibrium, and promoted oxidative conditions conducive to dormancy release. Accordingly, transgenic tubers expressing the acetylation-mimic StPRX3^K37Q^ maintained higher peroxidase activity and prolonged dormancy; tubers expressing the deacetylation-mimic StPRX3^K37R^ exhibited increased ROS accumulation and premature sprouting (Fig. 8, G to L). These findings identify K37 acetylation as a central molecular switch that integrates StSIRT1 activity with peroxidase-mediated redox regulation to control potato dormancy.

In summary, this study establishes a redox-sensitive posttranslational mechanism that links NAM metabolism, lysine acetylation, and oxidative signaling in control of potato dormancy. In addition to advancing the understanding of tuber storage biology, our findings provide a conceptual framework for manipulation of plant dormancy via targeted modulation of acetylation status and redox homeostasis. These regulatory principles may extend to broader plant systems and offer strategies for optimization of crop storage, stress resilience, and metabolic stability through precise control of acetylation-dependent redox networks. While this study uncovers extensive acetylation events linked to potato dormancy and sprouting, it remains unknown how individual acetylation signals are integrated with other posttranslational modifications to regulate developmental transitions. Notably, proteins involved in ubiquitination, phosphorylation, and methylation were frequently acetylated, indicating that acetylation may function within a broader modification network rather than acting in isolation. Multiple hypotheses have been proposed to explain posttranslational modification cross-talk, including hierarchical control, feedback regulation, and condition-specific coordination, yet experimental evidence in the context of plant dormancy is limited. Future studies should therefore prioritize systematic analyses of multimodification interactions to refine current models of posttranslational regulation. This work will contribute to a deeper understanding of plant dormancy control and provide a theoretical basis for exploring analogous regulatory mechanisms in other plant species.

## MATERIALS AND METHODS

### Plant materials and growth conditions

Potatoes (*S. tuberosum* L. cv. *Atlantic*) were harvested during the month of September from a local field in Gansu, China (each potato weighed 300 ± 50 g). After they had been cured in darkness for 20 days, tubers were rinsed with running water to remove surface soil, surface sterilized in 2% sodium hypochlorite for 2 min, washed three times with distilled water, and air dried at room temperature for subsequent transient transformation. To simulate commercial storage conditions, potatoes were stored in a growth chamber under dark conditions at 20°C and 58% relative humidity. Sampling was performed every 6 days from 0 to 24 days, with five potatoes randomly collected at each time point. Holes (5-mm diameter) were made at the bud eye position using a punch, and tissue slices were trimmed to a uniform thickness of 1 cm. The liquid nitrogen frozen tissues were used for subsequent measurements of gene expression, enzyme activity, and H_2_O_2_ content. For tissue-specific analyses, samples were collected from the peel, pulp, buds, and pulp beneath the buds of harvested potatoes. NAM and sirtinol treatments were performed as previously described (*22*). All samples were immediately frozen in liquid nitrogen and stored at −80°C until use, unless otherwise specified. We used WT potato (*S. tuberosum* L. cv. *Atlantic*) to generate transgenic and mutant lines. Seedlings were grown in a climate-controlled chamber at 25°C under a 16-hour light/8-hour dark photoperiod, 65% relative humidity, and a light intensity of ∼250 μmol m^−2^ s^−1^ provided by light-emitting diode illumination. Tobacco (*Nicotiana benthamiana*) were grown under identical conditions and used at 4 weeks of age for subcellular localization, BiFC assays, LCAs, and in vivo acetylation assays.

### Phylogenetic and promoter analysis

To investigate evolutionary relationships between target proteins and homologs, the potato reference genome was downloaded from the Phytozome database. Hidden Markov model profiles of conserved domains (PF00141 and PF02146) were retrieved from the Pfam database and used to identify candidate PRX and SIRT family genes in potato using HMMER. In parallel, *Arabidopsis thaliana* PRX and SIRT protein sequences from TAIR were used for BLASTP searches against the potato genome to identify homologs. Genes identified by both HMMER and BLASTP were intersected as putative candidates. SMART, Pfam, and CD-Search were used to validate conserved domains and confirm final family members. Multiple sequence alignments were performed separately for PRX and SIRT protein families using MEGA 11. Phylogenetic trees were constructed using the maximum likelihood method. For promoter analysis, 2000-bp upstream regions of the *StPRX3* and *StSIRT1* coding sequences were retrieved from the National Center for Biotechnology Information database. Cis-acting regulatory elements were predicted using PlantCARE; the distribution and classification were visualized using TBtools.

### Measurement of sprouting rate

Sprouting rate was determined according to the previous method with minor modifications (*22*). Potato dormancy release was defined as the stage in which at least one bud reached a length of 2 mm. Sprouting rate (%) was calculated as the ratio of the number of sprouted eyes to the total number of eyes per potato.

### Total RNA extraction and qRT-PCR analysis

Total RNA was extracted from tuber tissues using the FastPure Plant Total RNA Isolation Kit (RC401, Vazyme Biotech) in accordance with the manufacturer’s instructions. Approximately 80 mg of frozen tissue was used per extraction. First-strand cDNA was synthesized from 1 μg of total RNA using the HiScript III All-in-One SuperMix Perfect for qPCR kit (R333, Vazyme). qRT-PCR was performed on a QuantStudio 3 Real-Time PCR System (Applied Biosystems, USA) using Taq Pro Universal SYBR qPCR Master Mix (Q712, Vazyme). The elongation factor 1-α (*EF-1*α) gene was used as the internal reference, and relative expression levels were calculated using the 2^–ΔΔ*C*t^ method. Primer sequences for target genes are listed in table S1.

### Western blot analysis

Proteins were extracted from 0.5 g of frozen potato tissue using lysis buffer containing 50 mM tris-HCl (pH 6.8), 9 M urea, 4.5% (w/v) SDS, and 7.5% (v/v) β-mercaptoethanol, supplemented with EDTA-free protease inhibitors. Protein concentrations were determined using a BCA Protein Assay Kit (PC0020, Solarbio). Equal amounts of protein were separated by 4 to 20% SDS–polyacrylamide gel electrophoresis (SDS-PAGE) and electrotransferred onto methanol-activated polyvinylidene fluoride membranes (IPVH00010, Millipore, USA). Membranes were blocked with 5% (w/v) nonfat milk in 1× tris-buffered saline plus Tween (TBST) for 1 hour at 25°C and then incubated overnight at 4°C with an anti–acetyl-lysine rabbit monoclonal antibody (1:1000 dilution; PTM-105RM, PTM Bio). After three washes with TBST, membranes were incubated with horseradish peroxidase (HRP)–conjugated anti-rabbit immunoglobulin G (IgG) secondary antibody (1:5000 dilution; AS014, ABclonal) for 1 hour at room temperature. Immunoreactive bands were visualized using an enhanced chemiluminescence substrate (FD8020, Fude Biological Technology) and captured using a chemiluminescence imaging system.

### Acetyl-proteomics

Label-free four-dimensional acetyl-proteomic analysis was conducted by OE Biotech (Shanghai, China). Potato tissues from the NTDS and NPDS groups at 0 and 24 days were collected for protein extraction using lysis buffer. Precipitated proteins were digested overnight with trypsin in ammonium bicarbonate. Acetylated peptides were enriched using the PTMScan Acetyl-Lysine Motif [Ac-K] Kit (#13416, Cell Signaling Technology, USA), desalted with Pierce C18 Spin Tips, and lyophilized at 4°C. Peptide separation and identification were performed on a high-resolution liquid chromatography-tandem MS system equipped with a C18 analytical column (25 cm × 75 μm inner diameter, 1.6 μm; IonOpticks) via gradient elution. Raw data were processed using MaxQuant with a precursor ion mass tolerance of 10 to 20 parts per million and fragment ion tolerance of 0.5 Da. Quality control procedures included removal of reverse sequences, potential contaminants, and nonacetylated peptides. Proteins, peptides, and sites exhibiting fold changes ≥ 1.2 or ≤ 1/1.2 were considered significantly different. Detailed omics results are provided in tables S2 to S5.

### Mutant construction

The coding sequence of *StPRX3* (accession M1A2Y4) was amplified from cDNA using 2× Phanta Max Master Mix (Dye Plus) (P525-01, Vazyme) and purified with the FastPure Gel DNA Extraction Mini Kit (DC301-01, Vazyme). The amplified fragment was ligated into the pNC-AEnTopo vector (NC007, NC Biotech) and transformed into *Escherichia coli* DH5α competent cells (TSC-C14, Tsingke Biotech). Positive clones were identified by colony PCR using 2 × T5 Super PCR Mix (Colony) (TSE005, Tsingke Biotech) and verified by sequencing (Suny, Zhejiang, China). The pNC-AEnTopo-StPRX3 plasmid was extracted using the Easy Plasmid Miniprep Kit (DR0201050, Suny) and stored at −20°C. Site-directed mutagenesis was performed to generate StPRX3^K37Q^ and StPRX3^K37R^ mutants. Point mutations were introduced into the pNC-AEnTopo-StPRX3 plasmid using the Mut Express II Fast Mutagenesis Kit V2 (C214, Vazyme) in accordance with the manufacturer’s instructions. After Dpn I digestion and product purification, recombinant pNC-AEnTopo-StPRX3^K37Q^ and pNC-AEnTopo-StPRX3^K37R^ plasmids were transformed into DH5α cells and screened by colony PCR. Positive clones were verified by sequencing; confirmed plasmids were purified and stored for subsequent analyses. Primers used for genotyping are listed in table S6.

### Subcellular localization

The coding sequences of StSIRT1, StPRX3, StPRX3^K37Q^, and StPRX3^K37R^, lacking stop codons, were cloned into the pCAMBIA1302-eGFP vector using the Nimble Cloning system (NC001, NC Biotech) to generate StSIRT1-eGFP, StPRX3-eGFP, StPRX3^K37Q^-eGFP, and StPRX3^K37R^-eGFP fusion constructs. The resulting plasmids were introduced into Forty-eight hours postinfiltration, enhanced GFP (eGFP) fluorescence was visualized using an ultrahigh-resolution confocal microscope (LSM 980, Zeiss, Germany) with excitation and emission wavelengths of 488 and 510 nm, respectively. Primer sequences are provided in table S6.

### Transient transformation in potato

The full-length coding sequences of *StSIRT1* and *StPRX3* were cloned into the pNC-Cam23FN vector under the control of the CaMV 35S promoter to construct overexpression constructs. In parallel, 300-bp fragments of the StSIRT1 or StPRX3 coding sequences were inserted into the pNC-Cam2304-RNAi vector to construct RNAi plasmids. All recombinant plasmids were transformed into GV3101 and used for transient transformation via leaf infiltration in *N. benthamiana* or vacuum infiltration of tubers (0.8 MPa for 20 min). Empty vectors were used as negative controls. Infiltrated potatoes and leaves were incubated in darkness at 26°C. For potato assays, 15 tubers were used per treatment. Primer sequences used for plasmid construction are listed in table S6.

### Construction of transgenic potatoes

Sterile potato tissue–cultured seedlings were sectioned into 0.8-cm stem segments and precultured in darkness for 3 days. *A. tumefaciens* strains harboring pNC-Cam2304-RNAi-StSIRT1, pNC-Cam23FN-StPRX3, pNC-Cam23FN-StPRX3^K37Q^, and pNC-Cam23FN-StPRX3^K37R^ were grown to an optical density at 600 nm (OD_600_) of 1.0; harvested by centrifugation at 8000*g*; and resuspended in infection buffer to an OD_600_ of 0.3. Stem explants were immersed in the bacterial suspension and gently agitated for 15 min. Explants were placed on cocultivation medium and incubated in darkness for 3 days, followed by transfer to selection medium at 25°C under a 16-hour light/8-hour dark photoperiod for 14 days. Explants were subsequently transferred to shoot induction medium and subcultured every 2 days until shoot formation. Individual shoots were moved to rooting medium. After 3 weeks, regenerated plantlets were acclimated to soil to obtain stable *stsirt1-rnai*, StPRX3-Flag, StPRX3^K37Q^-Flag, and StPRX3^K37R^-Flag overexpression transgenic potatoes. Genomic DNA was extracted from transgenic plants for PCR-based confirmation of positive transformants. Primer sequences used for plasmid construction are provided in table S6.

### Enzyme activity assay

Peroxidase activity was measured using a Peroxidase Activity Assay Kit (BC0095, Solarbio), in accordance with the manufacturer’s instructions. Catalase and superoxide dismutase activities were measured according to established protocols (*72*).

### Quantitative detection of H_2_O_2_ and O_2_^–^

Tissue beneath potato buds was sectioned into slices measuring 8 mm by 1 mm by 10 mm and subjected to in situ H_2_O_2_ detection using 3,3′-diaminobenzidine (DAB) staining. Slices were incubated in DAB-tris-acetate buffer (0.1 mg ml^−1^; pH 5.0) at room temperature in darkness for 30 min and then sequentially rinsed with 70% ethanol and sterile water for 1 min each. Samples were subsequently photographed under natural light. Quantitative determination of H_2_O_2_ and O_2_^–^ contents was performed using Hydrogen Peroxide Content Assay Kit (BC3595, Solarbio) and Superoxide Anion Activity Content Assay Kit (BC1295, Solarbio), respectively.

### Determination of solanine content

For solanine quantification, 1 g of precooled tissue was suspended in 10 ml of 0.2% methanol-water solution and sonicated for 40 min. The mixture was incubated on a shaker at 4°C for 2 hours and subsequently centrifuged at 5000*g* for 10 min. The supernatant was filtered through a 0.22-μm membrane into brown high-performance liquid chromatography (HPLC) vials. Solanine content was analyzed using an HPLC system (e2695; Waters, USA) equipped with a C18 column (150 × 4.6 mm, 5 μm). The mobile phase consisted of phosphate buffer (pH 7.6) and acetonitrile [60:40 (v/v)] at a flow rate of 1 ml min^−1^. Solanine was detected at 220 nm. Standard curves were generated using solanine standards (Sigma-Aldrich, USA).

### Plant SIRT1 content assay

Plant SIRT1 content was determined using a SIRT1 ELISA Detection Kit (CB12127-Pt, Coibo Bio).

### LCA assay

pNC-Cam1300-Cluc-StPRX3, pNC-Cam1300-Cluc-StPRX3^K37Q^, pNC-Cam1300-Cluc-StPRX3^K37R^, and pNC-Cam1300-Nluc-StSIRT1 constructs were generated by amplifying the coding sequences of StPRX3, StPRX3^K37R^, StPRX3^K37Q^, and *StSIRT1*—without stop codons—and assembling them into the corresponding pNC-Cam1300-Cluc and pNC-Cam1300-Nluc vectors (NC Biotech) using the Nimble Cloning system. Verified recombinant plasmids were introduced into GV3101 (pSoup-p19). For split-luciferase assays, *Agrobacterium* cultures carrying Cluc-StPRX3^K37R^, Cluc-StPRX3^K37Q^, or Cluc-StPRX3 were mixed with Nluc-StSIRT1 at a 1:1 ratio and coinfiltrated into *N. benthamiana* leaves, ensuring nonoverlapping infiltration areas. At 48 hours postinfiltration, 1 mM d-luciferin was applied to the abaxial leaf surface, and bioluminescence was recorded using an imaging system (NightSHADE LB 985, Berthold Technologies, Bad Wildbad, Germany). Relative luciferase activity was quantified using a firefly luciferase assay kit (RG005, Beyotime) after protein extraction from infiltrated tissues.

### Y2H assay

The fusion constructs pNC-GBKT7-StPRX3, pNC-GBKT7-StPRX3^K37R^, pNC-GBKT7-StPRX3^K37Q^, and pNC-GADT7-StSIRT1 were generated by inserting the full-length coding sequences of StPRX3, StPRX3^K37R^, StPRX3^K37Q^, and *StSIRT1* into the GAL4 DNA binding domain vector pNC-GBKT7 and the activation domain vector pNC-GADT7, respectively, using the Nimble Cloning system (NC Biotech). The resulting BD and AD constructs were cotransformed into the *Saccharomyces cerevisiae* Y2HGold strain using a Y2HGold-GAL4 Yeast Two-Hybrid Interaction Validation Kit (YH2011; Coolaber). Transformants were plated on SD/−Leu/−Trp and SD/−Ade/–His/−Leu/−Trp media and incubated at 30°C for 96 hours. Single colonies were subsequently transferred to SD/−Ade/–His/−Leu/−Trp medium to assess protein-protein interactions.

### BiFC assay

For BiFC assay, the coding sequences of StPRX3 and StSIRT1 without stop codons were cloned into pNC-BiFC-Ecn and pNC-BiFC-Enn vectors (NC Biotech) to generate fusions with split yellow fluorescent protein (YFP) fragments. The resulting constructs were transformed into GV3101 (pSoup-p19); EVs served as negative controls. *A. tumefaciens* cultures harboring Enn-StSIRT1 and Ecn-StPRX3 were mixed and coinfiltrated into *N. benthamiana* leaves. At 48 hours postinfiltration, YFP fluorescence was visualized using a confocal microscope (excitation/emission: 488 nm/520 to 540 nm; LSM 980, Zeiss).

### Co-IP assay

For in vivo Co-IP assay, GV3101 strains carrying pNC-Cam23FN-StPRX3 and pNC-Cam1304-SubC-StSIRT1 were mixed at a 1:1 ratio and coinfiltrated into *N. benthamiana* leaves; EVs served as controls. After 48 hours, 0.5 g of infiltrated leaf tissue was collected, ground in liquid nitrogen, and resuspended in 1.2 ml of IP buffer [50 mM tris-HCl (pH 7.5), 150 mM NaCl, 5 mM EDTA, 0.1% Triton X-100, 5 mM dithiothreitol (DTT), 1 mM phenylmethylsulfonyl fluoride (PMSF), 2% glycerol, 5 mM MgCl_2_, and 100 μM EDTA-free protease inhibitor]. An aliquot (50 μl) of GFP-tagged soluble protein was mixed with 6× SDS loading buffer and boiled to serve as the input. The remaining soluble protein was incubated with anti-GFP magnetic beads (P2132, Beyotime) for 3 hours at 4°C with rotation. Beads were washed four times with cold IP buffer; bound proteins were eluted by boiling in 6× SDS loading buffer for subsequent Western blot analysis. Interactions between Flag-StPRX3 and GFP-StSIRT1 were detected using rabbit anti-Flag tag (AE004, ABclonal) and rabbit anti-GFP (AE078, ABclonal) primary antibodies, followed by HRP-conjugated goat anti-mouse (AS003, ABclonal) and anti-rabbit IgG (AS014, ABclonal) secondary antibodies.

### Expression of recombinant proteins in *E. coli*

Recombinant GST-StPRX3, GST-StPRX3^K37R^, GST-StPRX3^K37Q^, and His-StSIRT1 proteins were expressed in *E. coli* BL21(DE3) (EC1002, Weidi Biotech). Cultures were grown in LB medium at 37°C with shaking at 220 rpm until the OD_600_ reached 0.6. Protein expression was induced with 0.2 mM isopropyl-β-d-thiogalactopyranoside and continued at 16°C for 16 hours. Cells were then harvested by centrifugation. Bacterial pellets were resuspended in lysis/wash buffer [2 mM DTT, 1 mM PMSF, lysozyme (0.4 mg ml^−1^), ribonuclease A (10 μg ml^−1^), and deoxyribonuclease A (5 μg ml^−1^; pH 7.4)], lysed by sonication on ice, and centrifuged to remove insoluble material. The supernatant was filtered through a 0.22-μm membrane before purification. GST-StPRX3, GST-StPRX3^K37R^, and GST-StPRX3^K37Q^ were purified using the GSTPur Glutathione Kit (SA008K05, Smart-Lifesciences Biotech). His-SIRT1 was purified using the HisPur Ni-NTA Kit (SA004K03, Smart-Lifesciences Biotech). Purified proteins were analyzed by SDS-PAGE, concentrated to 1 mg ml^−1^ in phosphate-buffered saline containing 5% glycerol, and stored at −80°C.

### Pull-down assay

For in vitro pull-down assay, GST-StPRX3 or GST alone was preincubated with magnetic beads conjugated to rabbit anti-GST tag monoclonal antibody (AE122, ABclonal) in phosphate buffer (pH 7.4) at 4°C for 6 hours. After extensive washing to remove nonspecifically bound proteins, the beads were incubated with His-StSIRT1 overnight at 4°C. Bound proteins were eluted using 50 mM tris-HCl containing 10 mM reduced glutathione. Interactions between GST-StPRX3 and His-StSIRT1 were detected by Western blotting using rabbit anti-His tag (AE086, ABclonal) and rabbit anti-GST tag (AE077, ABclonal) primary antibodies, followed by HRP-conjugated goat anti-rabbit IgG (AS014, ABclonal) as the secondary antibody

### Acetylation assay

Western blot analysis was performed to assess acetylation levels of recombinant GST-StPRX3, GST-StPRX3^K37Q^, and GST-StPRX3^K37R^ proteins. Acetylation was detected using an anti–acetyl-lysine rabbit monoclonal antibody (PTM-105RM, PTM Bio). GST-tagged proteins were monitored using rabbit anti-GST tag antibody (AE077, ABclonal); HRP-conjugated goat anti-rabbit IgG (AS014, ABclonal) served as the secondary antibody.

### Structural modeling and molecular docking

Three-dimensional structures of StPRX3 and StSIRT1 were obtained from UniProt (www.uniprot.org/) in Protein Data Bank format and subjected to molecular docking via the HDOCK server (http://hdock.phys.hust.edu.cn/). The resulting docking complexes were visualized using PyMOL (Schrödinger, LLC, USA), and the model with the highest docking score was selected for subsequent analyses.

### In vitro deacetylation assay

For in vitro deacetylation assays, 50 ng of purified pGEX-4 T-StPRX3 or pGEX-4 T-StPRX3^K37Q^ was mixed with 50 ng of pNC-ET28-SIRT1 in NAD^+^-KDA buffer [100 μM NAD^+^, 50 mM NaCl, 0.1 mM EDTA, 0.2 mM PMSF, 5% glycerol, and 50 mM tris-HCl (pH 7.5)] and incubated at 30°C for 1 hour. Reactions were terminated through the addition of 6× SDS loading buffer, followed by boiling for 10 min. Deacetylation of GST-StPRX3 and GST-StPRX3^K37Q^ by His-StSIRT1 was evaluated by Western blotting. Primary antibodies were anti–acetyl-lysine rabbit monoclonal antibody (PTM-105RM, PTM Bio), rabbit anti-His tag (AE086, ABclonal), and rabbit anti-GST tag (AE077, ABclonal). HRP-conjugated goat anti-rabbit IgG (AS014, ABclonal) served as the secondary antibody.

### In vivo deacetylation assay

For in vivo deacetylation assay, *Agrobacterium* strains carrying pNC-Cam1304-SubC-StSPRX3, pNC-Cam1304-SubC-StPRX3^K37Q^, or pNC-Cam1304-SubC-StPRX3^K37R^ were mixed at a 1:1 volume ratio with strains carrying pNC-Cam23FN-StSIRT1. pNC-Cam23FN EV was used as the control. Mixtures were incubated at room temperature in darkness for 2 hours before infiltration. *N. benthamiana* leaf veins were infiltrated on opposite sides with mixtures containing pNC-Cam23FN-StSIRT1 × pNC-Cam1304-SubC-StPRX3^K37R/Q^ (experimental group) and pNC-Cam23FN × pNC-Cam1304-SubC-StPRX3^K37R/Q^ (control group), ensuring nonoverlapping infiltration zones. After 48 hours, 0.5 g of leaf tissue from each group was harvested and ground to a fine powder. Total protein was extracted using lysis buffer and incubated with 30 μl of preequilibrated anti-Flag magnetic beads (P2115, Beyotime) at 4°C in darkness for 4 hours. Beads were washed four times with 2 ml of precooled IP buffer for 10 min each. Deacetylation of GFP-StPRX3, GFP-StPRX3^K37Q^, and GFP-StPRX3^K37R^ by Flag-StSIRT1 was assessed by Western blotting. Primary antibodies were anti–acetyl-lysine rabbit monoclonal antibody (PTM-105RM, PTM Bio), rabbit anti-Flag tag (AE004, ABclonal), and rabbit anti-GFP tag (AE078, ABclonal). HRP-conjugated goat anti-rabbit IgG (AS014, ABclonal) served as the secondary antibody.

### Physicochemical properties of proteins

Physicochemical properties and structural features of StPRX3 and StSIRT1 were predicted using ProtParam (https://web.expasy.org/protparam/). Secondary structure was assessed via SOPMA (https://npsa-prabi.ibcp.fr/cgi-bin/npsa_automat.pl?page=npsa_sopma.html). Three-dimensional models of StPRX3 and StSIRT1 were generated with SWISS-MODEL (https://swissmodel.expasy.org/). Hydrophobicity profiles were predicted using ProtScale (https://web.expasy.org/protscale/), signal peptides were identified with SignalP 6.0 (https://services.healthtech.dtu.dk/services/SignalP-6.0/), and transmembrane regions were analyzed using TMHMM 2.0 (https://services.healthtech.dtu.dk/services/TMHMM-2.0/).

### Statistical analysis

All experiments were conducted with at least three independent biological and technical replicates. Data are presented as mean ± SD. Statistical significance was determined using Student’s *t* test, one-way analysis of variance (ANOVA) followed by Tukey’s post hoc test, or two-way ANOVA followed by Tukey’s multiple-comparison test. Data analysis and graphical presentation were performed using GraphPad Prism 9.0. Significant differences were indicated by asterisks and (*P < 0.05; **P < 0.01; ***P < 0.001). Figures 1 (H and J) and 9 and fig. S6A were created using BioRender and were licensed under CC BY 4.0 (BioRender.com/1nhkapo; BioRender.com/4mp2r2v; BioRender.com/fd5wjur). Protein quantification analysis was quantified using ImageJ, and all Western blots were independently repeated three times.
