# Supplementary material for: StSIRT1 regulates potato dormancy through deacetylation of peroxidase-like 3 at K37
Source: Sci Adv. 2026 Oct 28;12(44):eaef7735. doi: 10.1126/sciadv.aef7735 (PMC13715716; doi:10.1126/sciadv.aef7735)
Supplement: Supplementary file 1 [file sciadv.aef7735_sm.pdf]

Science Advances
AAAS

Supplementary Materials for

StSIRT1 regulates potato dormancy through deacetylation of peroxidase-like 3 at K37

Qiannan Hu *et al.*

Corresponding author: Zisheng Luo, luozisheng@zju.edu.cn; Li Li, lili1984@zju.edu.cn

Sci. Adv. **12**, eaef7735 (2026)
DOI: 10.1126/sciadv.aef7735

This PDF file includes:

Figs. S1 to S15
Tables S1 to S6

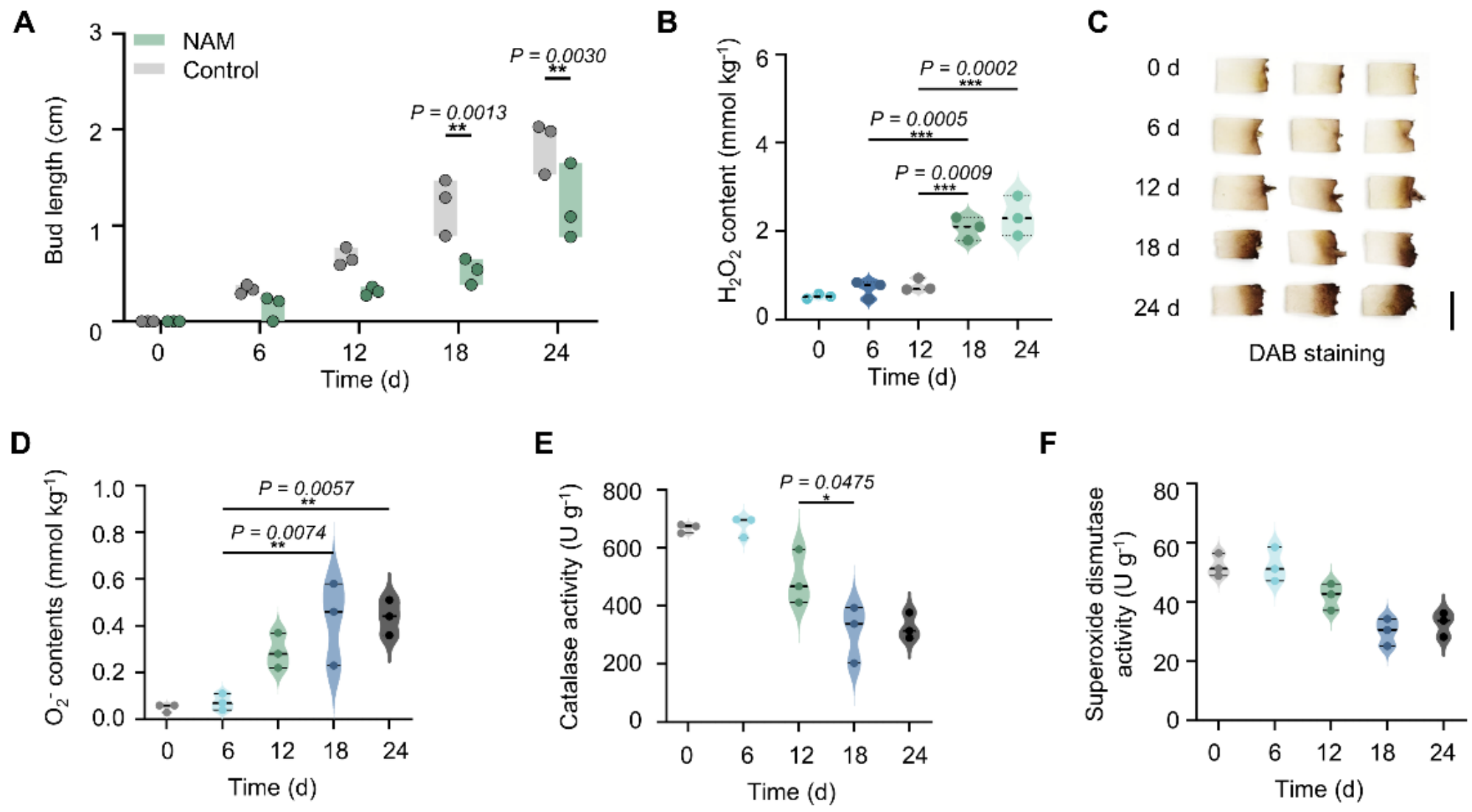

Fig. S1. Redox dynamics during potato dormancy and sprouting. (**A**) Bud length of NAM-treated potatoes during the storage. (**B**) H_2O_2 content of potatoes during storage from 0 to 24 d. (**C**) DAB staining of potatoes during storage from 0 to 24 d. Scale bar, 1 cm. (**D**) O_2- content in potatoes during storage from 0 to 24 d. (**E**) Catalase activity in potatoes during storage from 0 to 24 d. (**F**) Superoxide dismutase in potatoes during storage from 0 to 24 d. Values are means ± SE, n = 3 for [(A) to (F)]. One-way ANOVA with Tukey's test was used in (B, D to F); Two-way ANOVA with Šídák's multiple comparisons test was used in (A). *$P < 0.05$, **$P < 0.01$, *** $P < 0.001$.

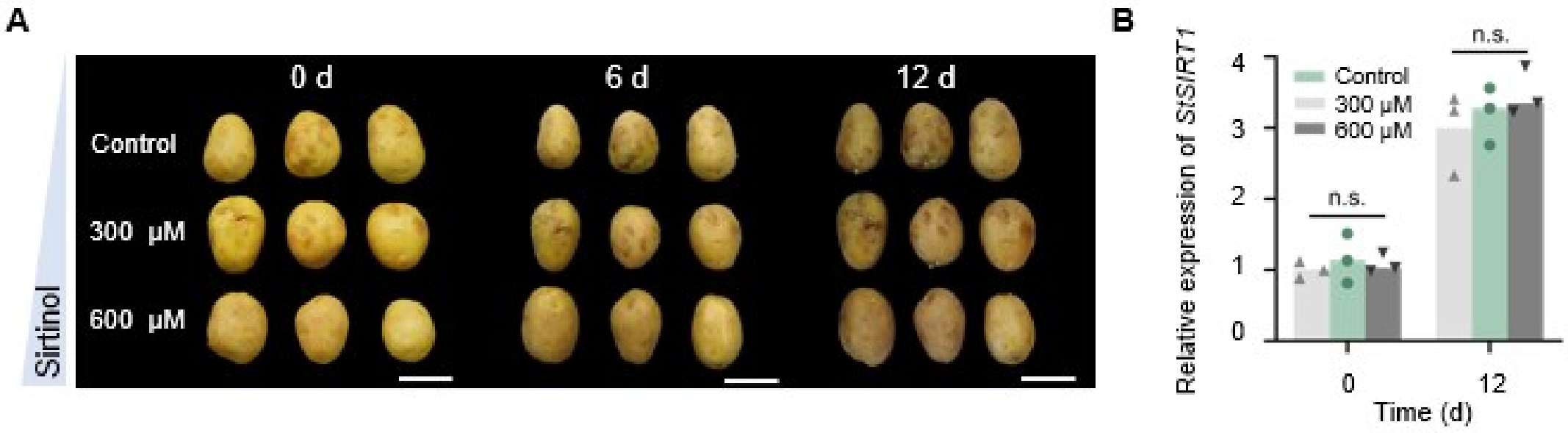

Fig. S2. Effects of sirtinol on potato dormancy. (**A**) Phenotypes of potatoes treated with sirtinol (0, 300, 600 μM). Potatoes were stored under dark conditions at 20 °C for 12 d, with images recorded every 6 d. Scale bar, 5 cm. (**B**) Expression levels of *StSIRT1* in response to sirtinol in potatoes; *EF-1α* was used as an internal control. Values are means ± SE, n = 3 for [(A) to (B)]. Two-way ANOVA with Šídák's multiple comparisons test was used in (B). n.s. represents not significant.

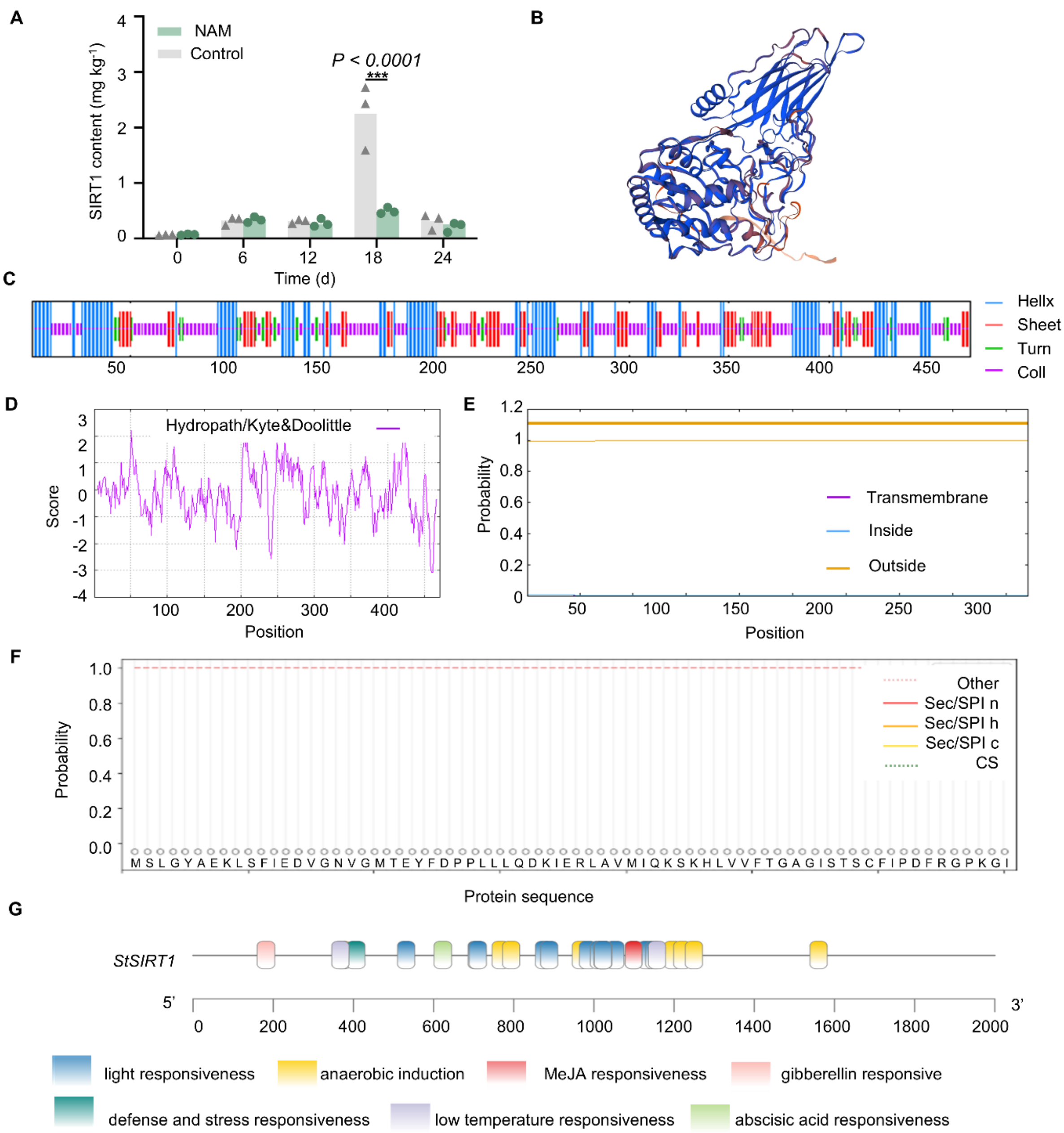

Fig. S3. Prediction and analysis of physicochemical properties of StSIRT1. (**A**) StSIRT1 content in EV-OE, OE-StSIRT1, EV-RNAi, and RNAi-StSIRT1 lines. (**B**) Tertiary structure model. (**C**) Secondary structure distribution. (**D**) Hydrophilicity and hydrophobicity analysis. (**E**) Transmembrane region prediction. (**F**) Signal peptide prediction. Phylogenetic analysis of potato StSIRT1 proteins. (**G**) Analysis of the StSIRT1 promoter region (2,000 bp upstream of the transcription start site). Values are means ± SE, n = 3 for (A). Two-way ANOVA with Šídák's multiple comparisons test was used in (A). *** $P < 0.001$.

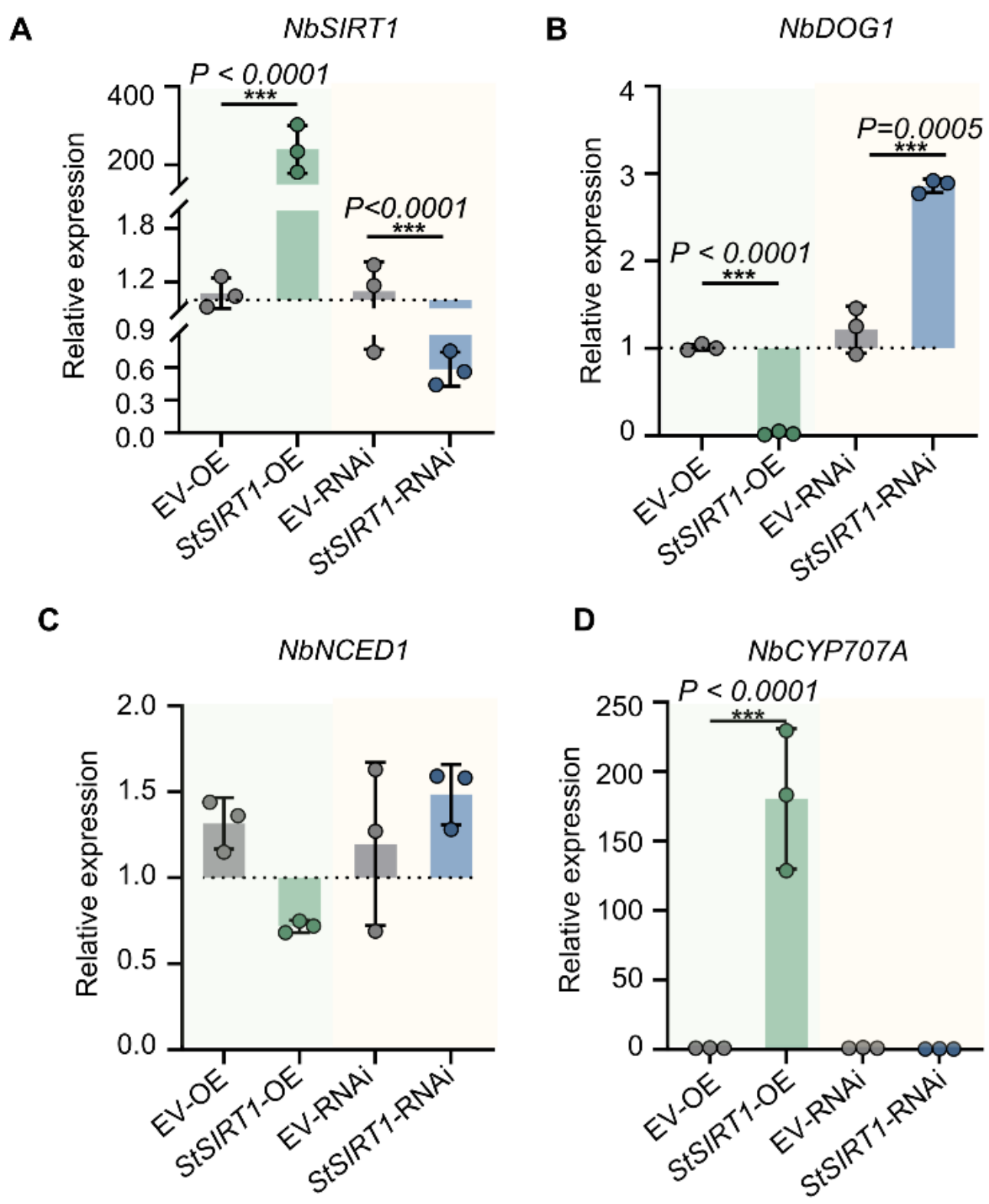

Fig. S4. Effects of heterologous expression of StSIRT in tobacco on dormancy-related marker genes. (**A** to **D**) Expression levels of *NbSIRT1*, *NbDOG1*, *NbNCED1*, and *NbCYP707A* in *N. benthamiana* leaves at 3 d; *EF-1α* was used as an internal control. Values are means ± SE, n = 3 for [(A) to (D)]. One-way ANOVA with Tukey's test was used for significant difference analysis. (C to F). *** $P < 0.001$.

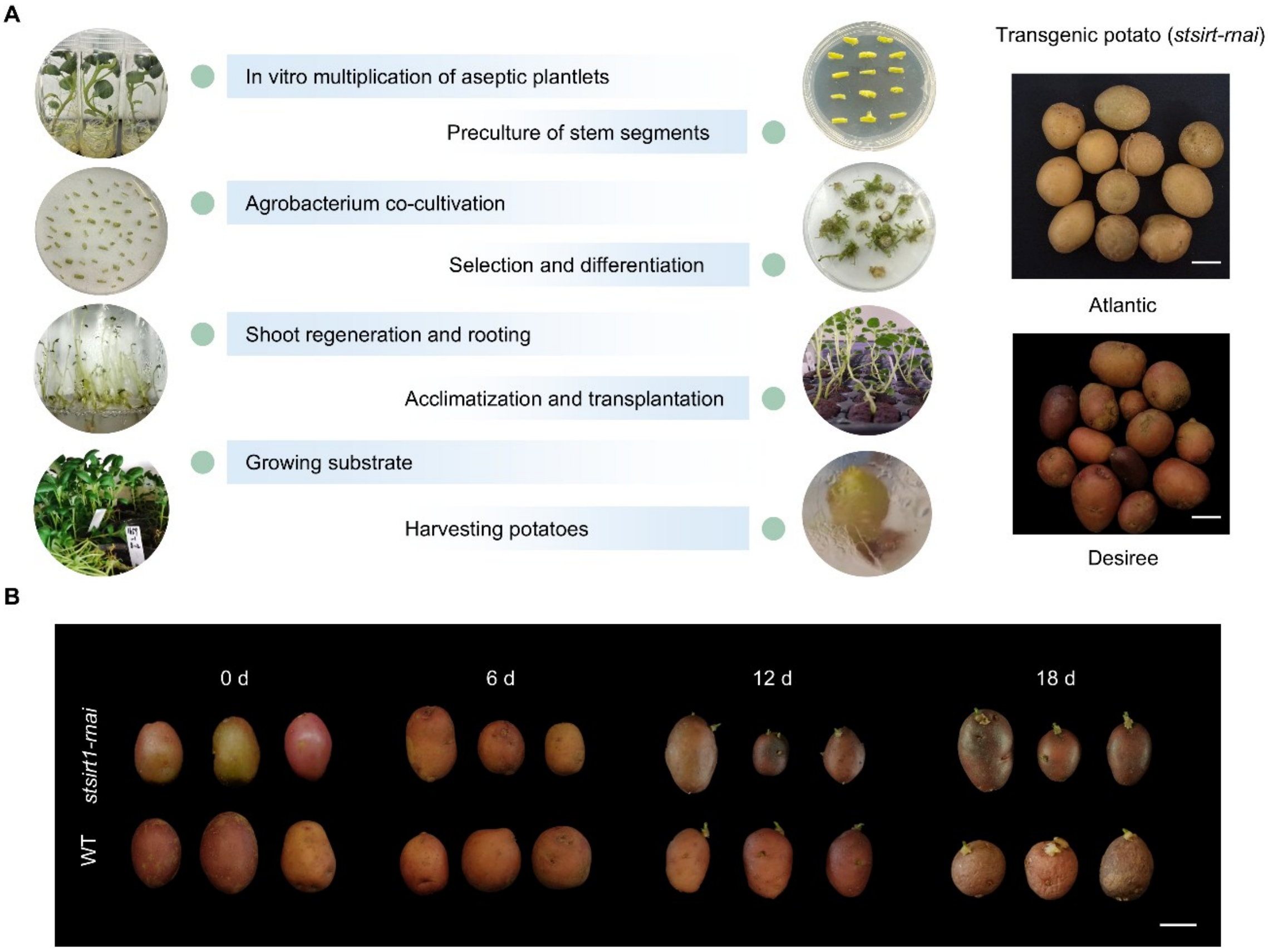

Fig. S5. Phenotypic of *stsirt-rnai* transgenic potatoes. (**A**) Genetic transformation procedure for *stsirt-rnai* transgenic potatoes. The cultivars used were '*Atlantic*' and '*Desiree*' (**B**) Phenotypes of stable transgenic *stsirt-rnai* '*Desiree*' potatoes. WT lines served as controls. Potatoes were stored under dark conditions at 20 °C for 18 d, with images recorded every 6 d. Scale bar, 1 cm.

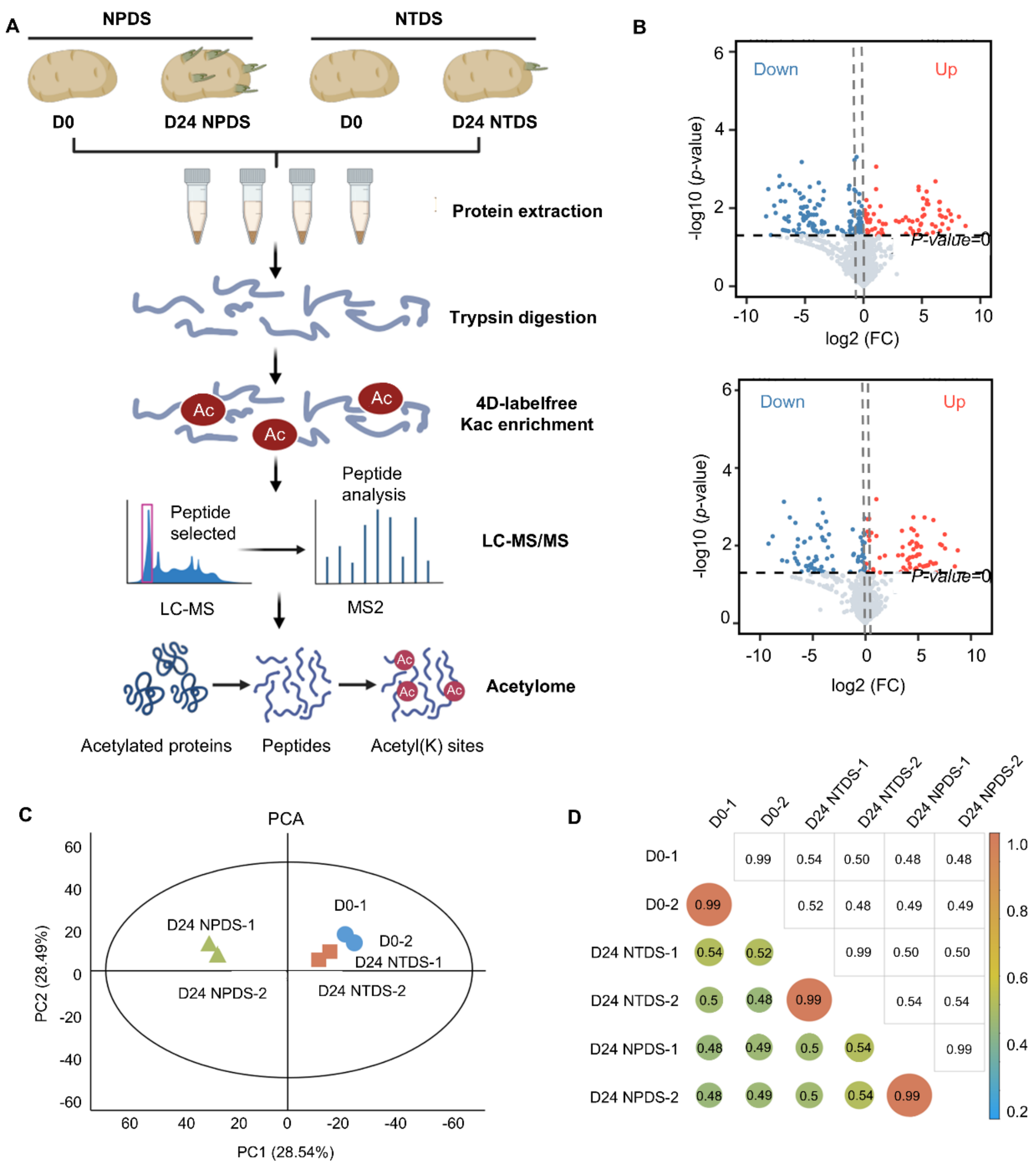

Fig. S6. Quality control of four-dimensional (4D) label-free acetyl-proteomic analyses. (**A**) Sample grouping and 4D label-free acetyl-proteomic workflow; "Ac" indicates acetylation. (**B**) Volcano plot showing upregulated (red) and downregulated (blue) acetylated proteins in NTPS and NTDS samples. (**C**) Principal component analysis of biological replicates for NPDS, NTDS, and D0 samples. (**D**) Correlation analysis among biological replicates of NPDS, NTDS, and D0 samples. Fig. S6A was created using BioRender and was licensed under CC BY 4.0.

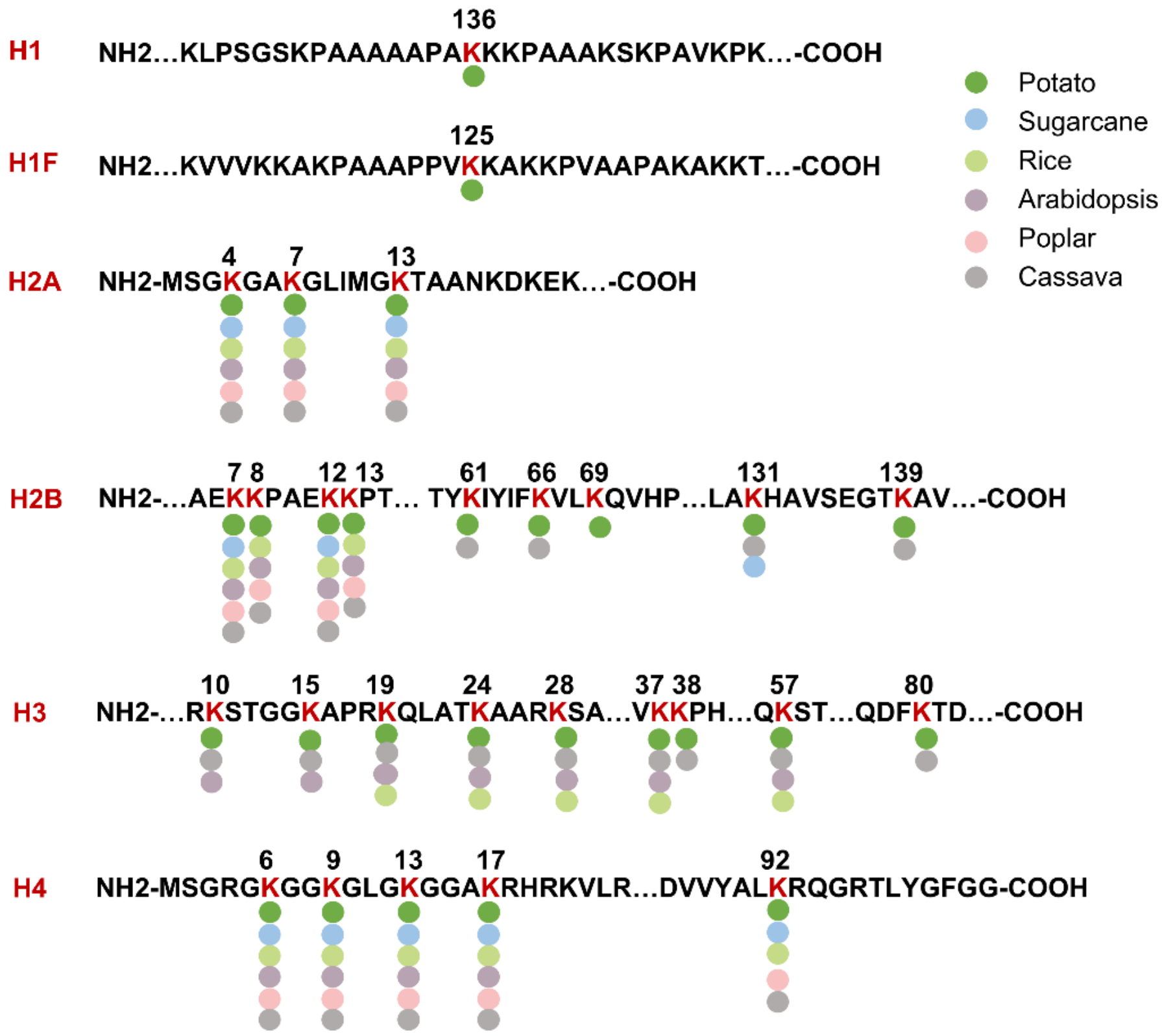

Fig. S7. The comprehensive atlas of histone acetylation in potato dormancy release. Histone lysine acetylation sites identified in potato and corresponding modifications in *Saccharum* spp. hybrids (Sugarcane), *Oryza sativa* (Rice), *Arabidopsis thaliana* (*Arabidopsis*), *Populus tremula* × *P. alba* (Poplar), and *Manihot esculenta* Crantz (cassava).

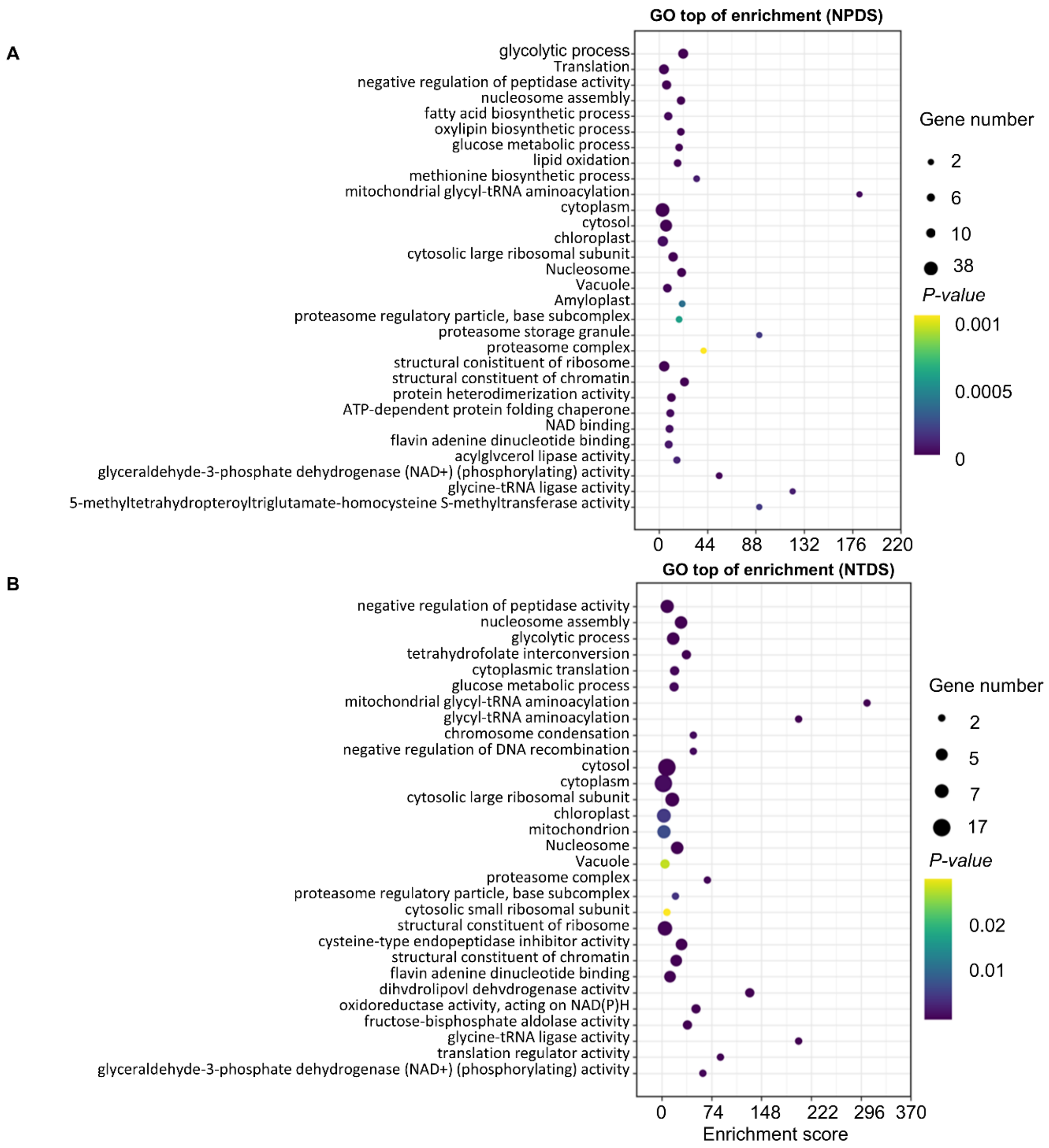

Fig. S8. GO enrichment analysis of lysine-acetylated proteins. (**A**) GO enrichment in the NPDS group. (**B**) GO enrichment in the NTDS group. Each circle denotes a GO term; circle size indicates the number of clustered acetylated proteins, and circle colour indicates enrichment significance.

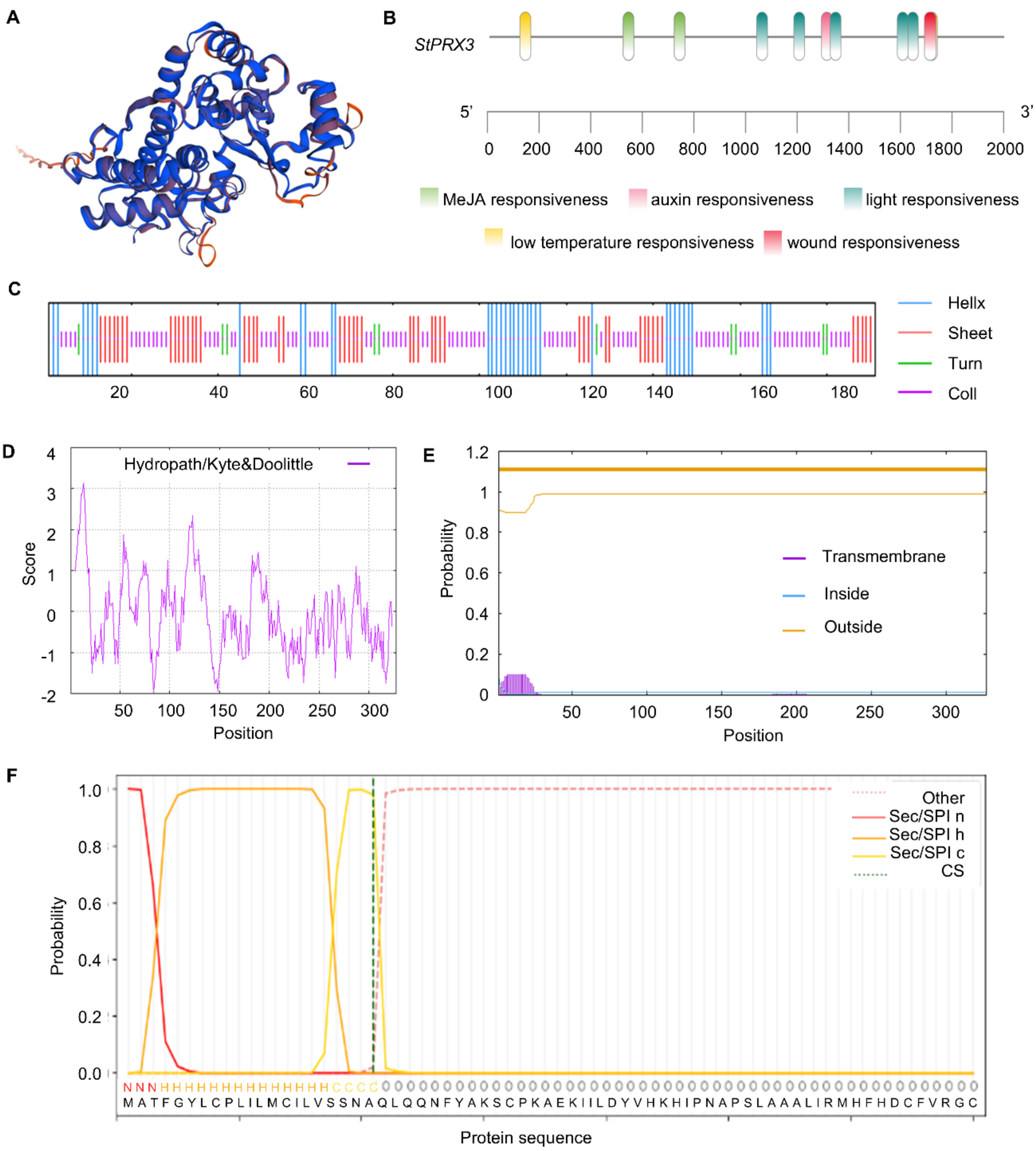

Fig. S9. Prediction and analysis of physicochemical properties of StPRX3. (**A**) Tertiary structure model. (**B**) Analysis of the promoter region (2,000 bp upstream) of potato *StPRX3*. (**C**) Secondary structure distribution. (**D**) Hydrophilicity and hydrophobicity analysis. (**E**) Transmembrane region prediction. (**F**) Signal peptide prediction.

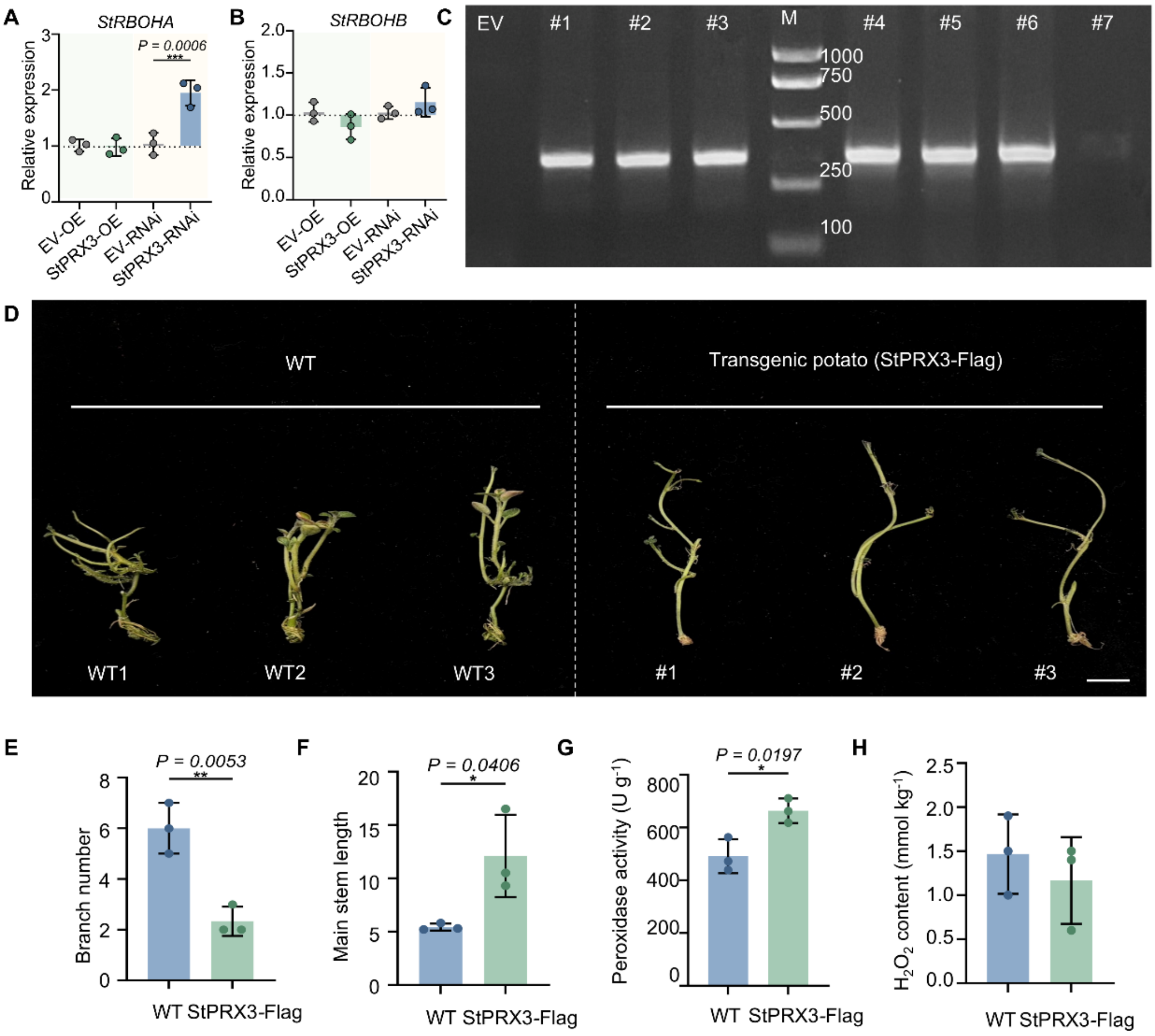

Fig. S10. Phenotypic and biochemical analyses of transgenic potato plants. (**A** to **B**) Expression levels of *StRBOHA and StRBOHB* in OE-StPRX3 and RNAi-StPRX3 potatoes at 24 d; *EF-1α* was used as an internal control. (**C**) qRT-PCR identification of transgenic plants. EV, positive control; #1–#7, StPRX3 transgenic lines; M, DL1000 marker. (**D**) Plant phenotype. (**E**) Branch number and (**F**) main stem length of StPRX3-Flag plants. (**G**) Peroxidase activity and (**H**) H_2O_2 content in StPRX3-Flag plants. Values are means ± SE, n = 3 for [(A to B) and (E to H)]. One-way ANOVA followed by Tukey's test was used in (A to B); Student's t test (two-tailed) was used in (E to H). *$P < 0.05$, **$P < 0.01$, *** $P < 0.001$.

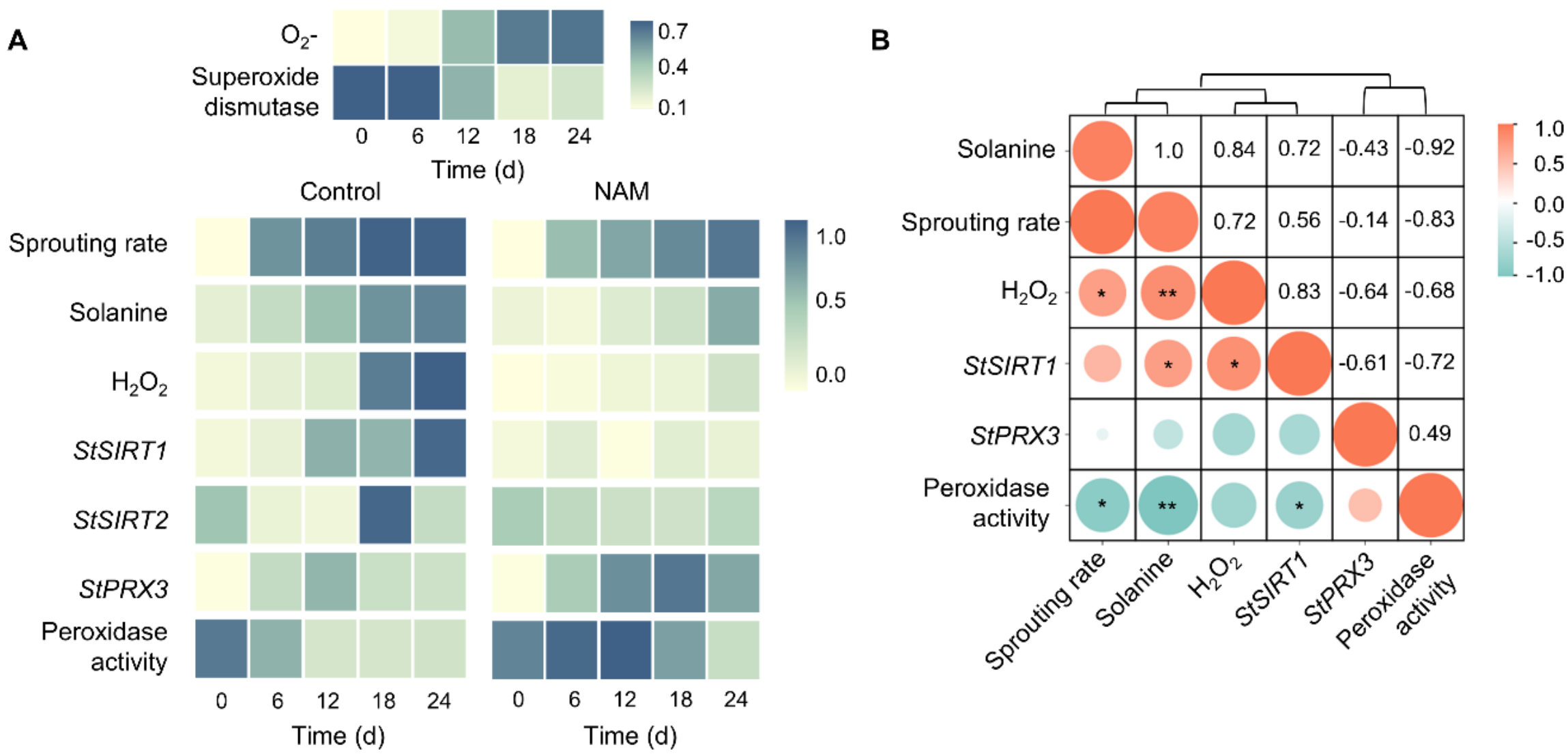

Fig. S11. StSIRT1-StPRX3-H_2O_2 regulatory axis in potato dormancy release. (**A**) Temporal heatmap of *StSIRT1*, *StPRX3*, antioxidant enzymes, ROS, solanine, and sprouting rate during potato dormancy release. (**B**) Correlation analysis of key regulators during potato dormancy release. Values are means ± SE. **P* < 0.05, ***P* < 0.01, *** *P* < 0.001.

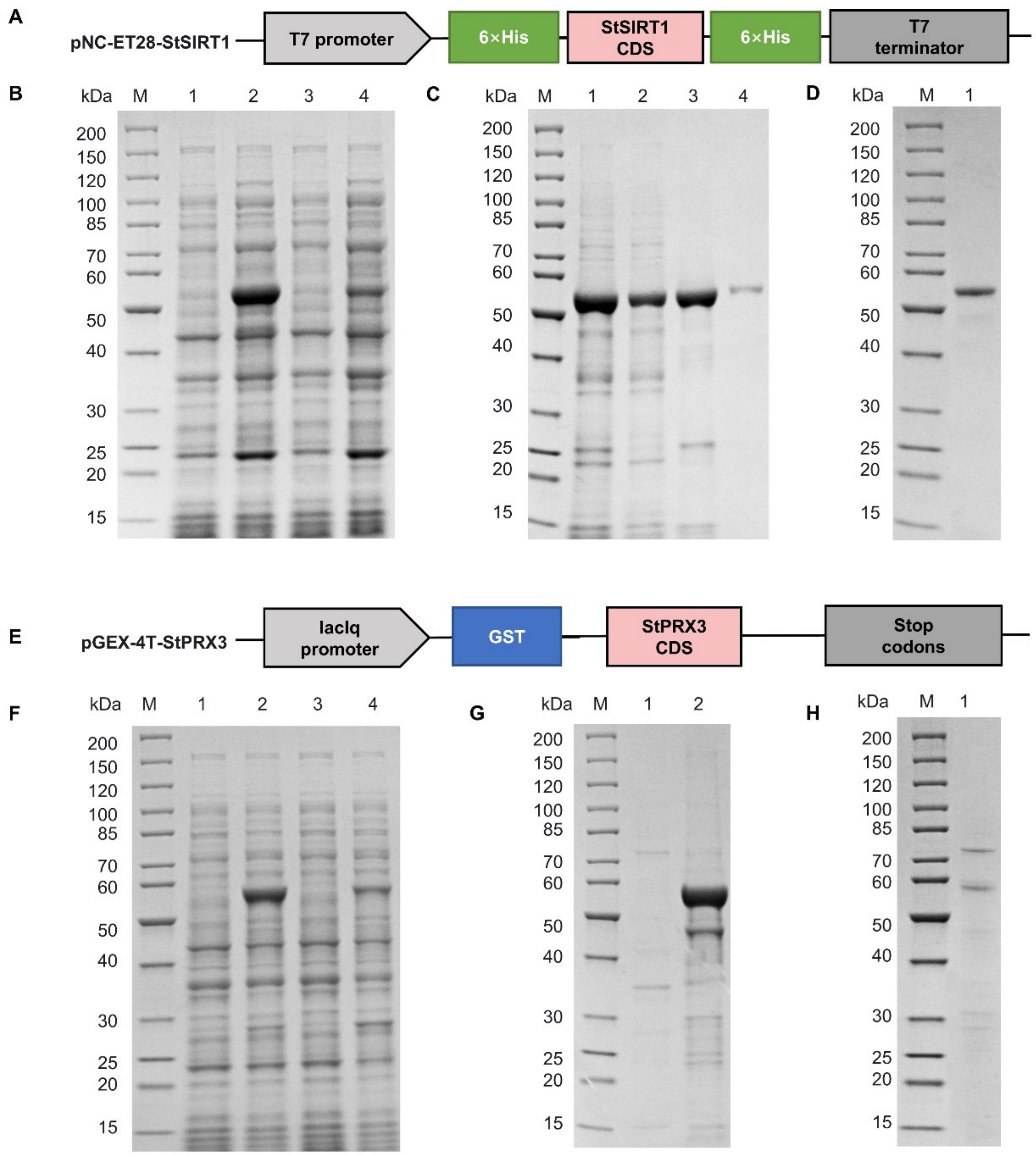

Fig. S12. SDS-PAGE analysis of purified recombinant StSIRT1-His and StPRX3-GST fusion proteins. (**A** and **E**) Schematic diagrams of vector construction. (**B**) SDS-PAGE analysis of StSIRT1-His expression optimisation (M, marker; lanes 1-2, before and after induction at 37°C; lanes 3-4, before and after induction at 16°C). (**C**) Affinity purification of StSIRT1-His (M, marker; lanes 1-2, elution fractions). (**D**) Concentration analysis of StSIRT1-His (M, marker; lane 1, purified protein). (**F** to **H**) SDS-PAGE analysis of StPRX3-GST expression and purification, with lane designations corresponding to panels (B to D).

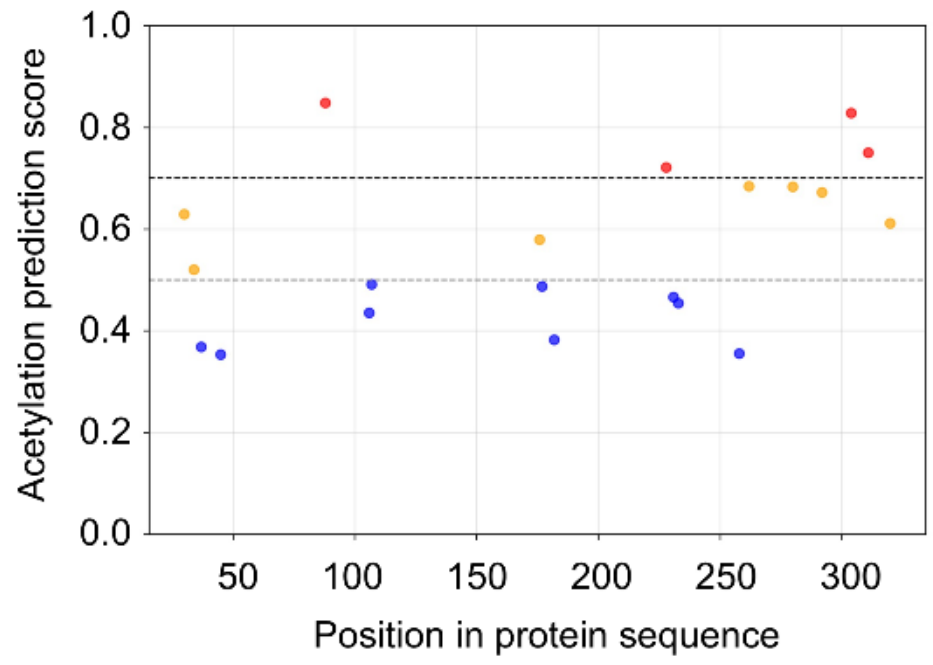

Fig. S13. Prediction of lysine acetylation sites in StPRX3 protein sequence. Red and orange dots indicate lysine acetylation sites with high predicted probability.

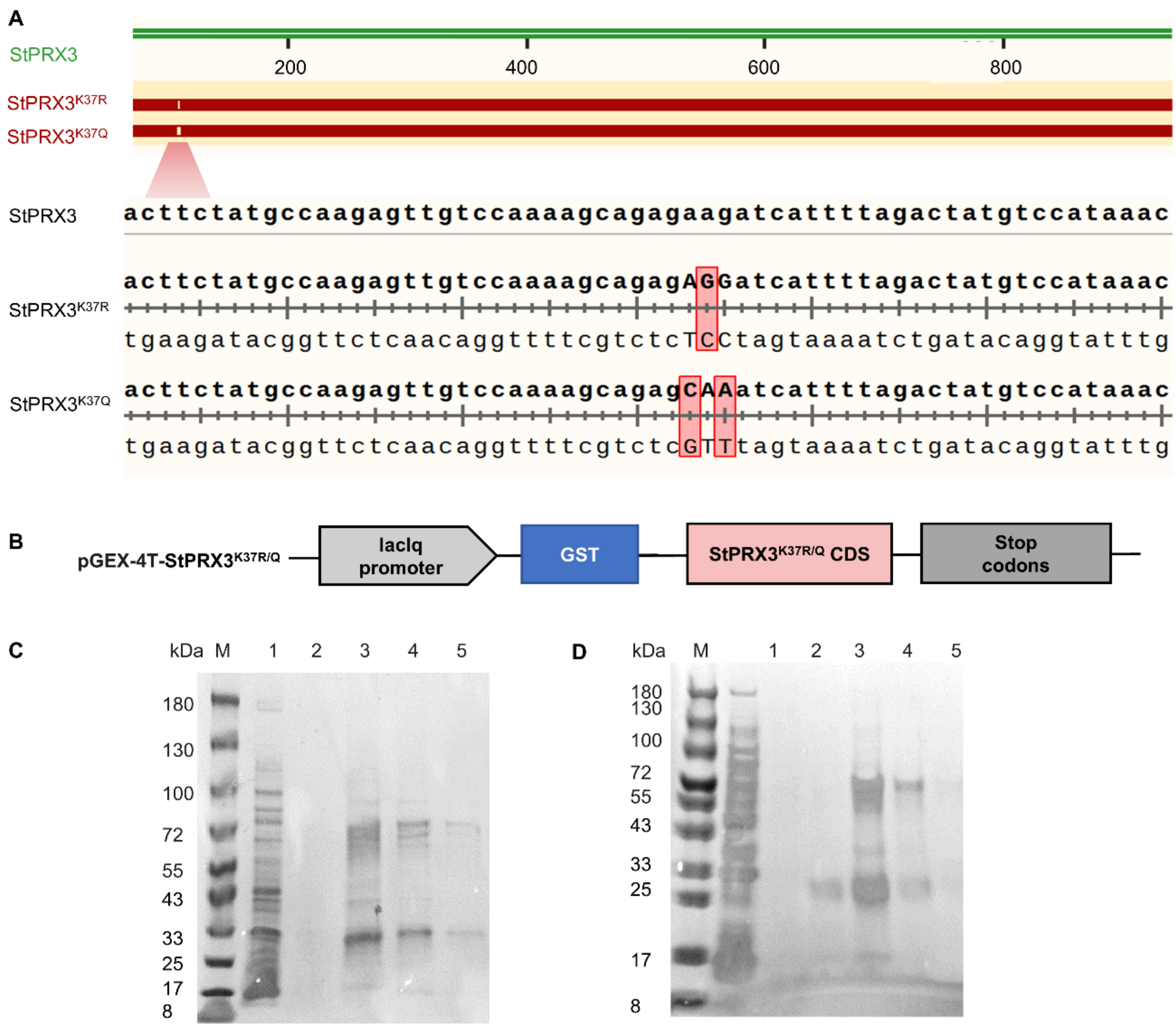

Fig. S14. SDS-PAGE analysis of purified recombinant StPRX3^{K37Q}-GST and StPRX3^{K37R}-GST mutants. (**A**) Point mutation strategy to simulate acetylation and deacetylation. The codon for K37 (AAG) in StPRX3 was replaced with codons encoding glutamine (CAA) or arginine (AGG) to generate the K37Q and K37R mutants, respectively. (**B**) Schematic diagram of vector construction. (**C**) Purification of GST-StPRX3$^{K37Q/R}$ proteins (M, marker; lane 1, flow-through; lanes 2-5, gradient elution fractions).

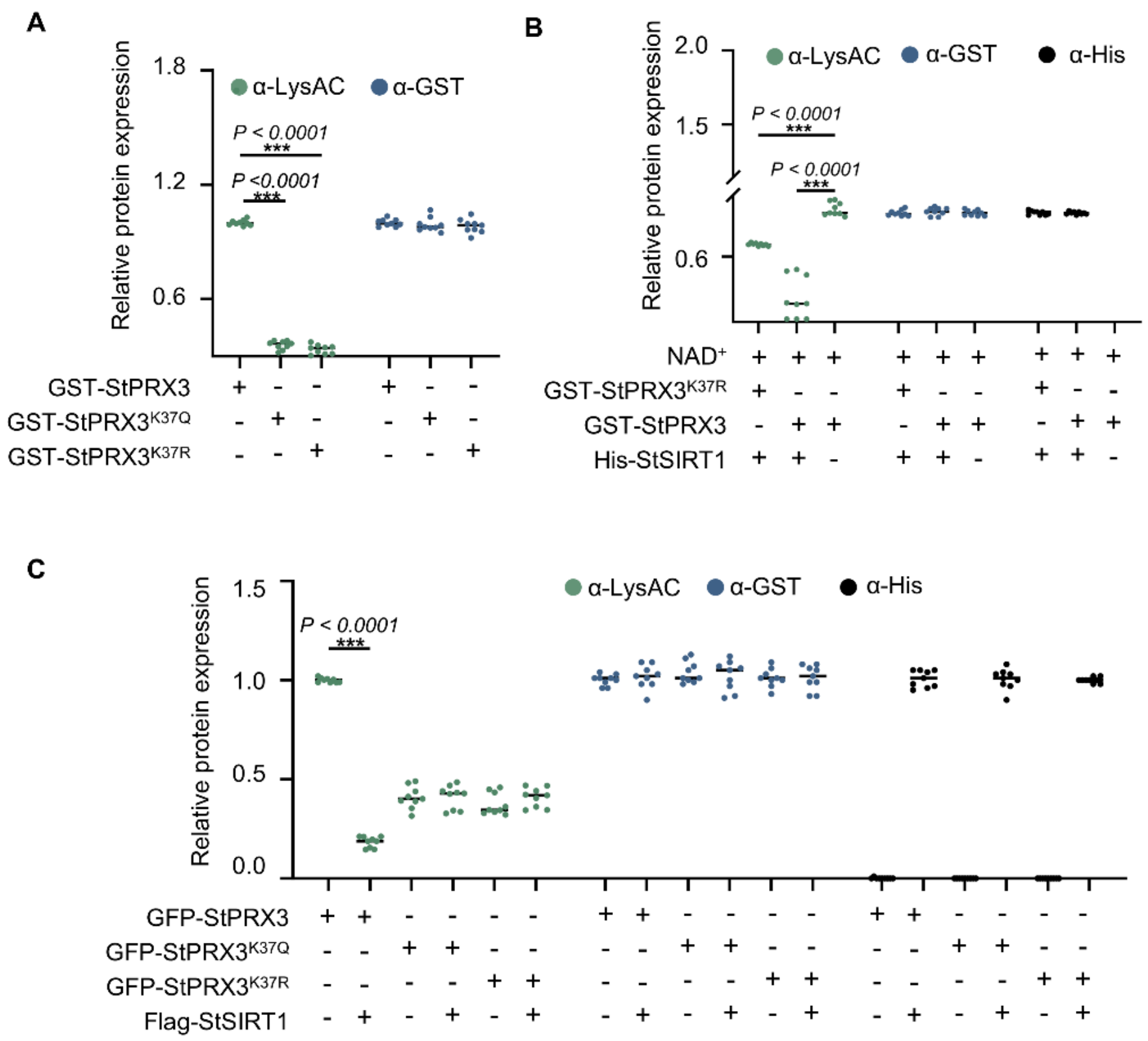

Fig. S15. Western blot quantification of StSIRT1 mediated deacetylation of StPRX3. (A) Acetylation levels of purified GST-StPRX3 and GST-StPRX3$^{K37R/Q}$. (**B**) Purified His-StSIRT1 deacetylated GST-StPRX3 and GST-StPRX3^{K37R} *in vitro*. (**C**) Flag-StSIRT1 deacetylates GFP-StPRX3 and GFP-StPRX3$^{K37R/Q}$ *in vivo*. Values are means ± SE, n = 9 for [(A to C)]. Two-way ANOVA with Šídák's multiple comparisons test was used in (A to C). *** $P < 0.001$.

Table S1. Primers used for RT-qPCR analysis.

Gene	Forward primer (5′–3′)	Reverse primer (5′–3′)
StEF-1α	ATTGGAAACGGATATGCTCCA	TCCTTACCTGAACGCCTGTCA
StDOG1	ATACATGGACACCCGCAGACA	TGCCACTCATCGCATAAAGGA
StABI5	ACGACAGACTACGTTCGGTGAGATG	CGGTTTGTGGAAGTGGCGGATAA
StCYP707A1	GTACAGGTGGTCTATGGTGG	CGATGCTTGTTGTCTTGATGTTGATT
StRBOHA	GATGCACGTTCAGCTCTCATTACCA	TTCTAAGCCATCAGACCAAAGCAGA
StRBOHB	ATCAAATGGAGATCGAAAACACGAG	TCAGACCATCCTTCAGTTCCTACAT
StSIRT1	CAGTTCTTGACTGGGAGGATG	GATCTGCAAACTTGTCCCTAGA
StPRX3	CAGACATTCCAGCACCAACTA	CCAATAGTGTGAGCACCAGATAA
StNCED1	ACAGCCGGACACCATTTCTT	CTAAACCGGCGTTTGCAACT
NbEF-1α	CGTCAAGAACGTTGCAGTTAAG	TGATGATGACTTGGGAGGTAAAG
NbSIRT1	GCGAGACTTAGAGACACAGTTC	TCCCAGACATAGCACAACATC
NbDOG1	GCAGGCGGTGAAGCTATTAG	CCACTGCGGATAAATTCCTAC
NbNCED1	CGACCCACGAGTCCAGATTTC	GAGCCTAGCAATTCCCGAGTG
NbCYP707A1	GAAGCTGTGGCTGATGTAGAA	AAGAACTCTGGGTTGTGATGAA
NbPRX3	TGGATCTCATACTGGTCTCA	TTCCATTGTTTCAGTCATTCA

Table S2. Differential acetylation sites between natural dormancy and sprouting groups (NPDS).

Accession	Positions	Acetyl (K) Probabilities	FC	Product
A0A8F6UAP5	136; 130	NGK(1)FVCEGESDEPK	0.33	Proteinase inhibitor II (Fragment)
A0A8F6UAP5	103;103;103;48;103;97;97	NCDTNIAYSK(1)CLR	0.42	Proteinase inhibitor II (Fragment)
A0A097H111	160;160;160	ADSSYFK(1)IVK	0.13	KTI-A protein
P58514	218;218;218;218;218;218	DNPLDISFK(1)QVQ	0.39	Serine protease inhibitor 1
A0A097H164	133;134;58;63;132; 132;135	TTK(1)LCVDQTVWK	0.50	KTI-C protein (Fragment)
A0A097H164	163;164;164	VGNENDIFK(1)IR	0.43	KTI-C protein (Fragment)
A0A1X9QDP2	88	NGK(1)FVCEGESDEPK	0.33	Proteinase inhibitor II (Fragment)
O04829	99;99;99;99	VK(1)LAVLQFYK	0.70	Ubiquitin extension protein
M0ZHQ9	304;304	VPNVTDIGLK(1)PR	0.45	3-hydroxyacyl-CoA dehyrogenase
M0ZNL5	52	SSELLQYNPVHK(1)K	0.83	Glutathione transferase
M0ZNL7	211	LVTK(1)YQLLHEK	0.75	Glutathione transferase
M1AY24	37	AEK(1)IILDYVHK	0.69	Peroxidase
P07745	367; 113;37; 368;113;367	DSPETYEEALK(1)R	0.50	Patatin
Q2MY52	277;277;277;277;278;23	TYTAQEAAK(1)WGPLR	0.57	Patatin group M-2
M1A5P9	115;115;300;262;293;21	IYETK(1)IWVK	0.47	Cysteine proteinase inhibitor
M0ZZR6	132;132	GCYYFGK(1)DGK	0.42	Proteinase inhibitor type-2 CEVI57
Q8LK04	337;337	VIDLICHMAK(1)A	0.78	Glyceraldehyde-3-phosphate dehydrogenase
M1A5P9	115;115;300;262;293;21	IYETK(1)IWVK	0.47	Cysteine proteinase inhibitor
M1AE23	130;130	ATSSSVDFQK(1)K	0.79	Protein Z
M1AG69	26	PAAEK(1)APVAAAEK(1)SK	0.72	Histone H2B
M1B647	142	LGK(1)LLDVYESR	0.70	Glutathione transferase
M1BEW2	23;23	PAAEK(1)APAEK(1)K	0.74	Histone H2B
M1BSE4	22	AGK(1)TTAAAAANK(1)EK	0.68	Histone H2A
Q09011	20;20	GK(1)LKPSSSSSFR	0.10	Calcium-binding protein CAST
O24388	101;106;177	TDLVTPGGSK(1)YVYK	0.38	Cysteine protease inhibitor 3
Q8W143	185	LLHCPSHLQCK(1)N	0.28	Putative Kunitz-type tuber invertase inhibitor (Fragment)
B2M0R3	196;196;196	WIK(1)NNGGNFR	2.27	Starch-granule-bound R1 protein
Q93XN9	126;130;134;134;87	GGHELSK(1)STGNAGGR	1.20	Superoxide dismutase (Fragment)
K7VPG7	43;43;43;43;43	VTGGAPNK(1)LSK	1.25	60S ribosomal protein L35
Q07511	79;79	GHQYIVTPDK(1)EGPDCELEK	3.56	Formate dehydrogenase, mitochondrial

Accession	Positions	Acetyl (K) Probabilities	FC	Product
M0ZK75	52;52	LDQAIENDK(1)K	1.43	Reverse transcriptase domain-containing protein
M0ZP30	80;83;83	MEIAADSLYK(1)AK	1.33	Pyruvate dehydrogenase E1 component subunit alpha
M0ZLC3	192;192	VWK(1)LYNGVWK	2.72	Protein transport SEC13
M0ZMA9	138	GDYVVSEEEHNVGK(1)GK	1.22	PR10
M0ZMF8	401	MVQLK(1)GHR	1.88	Phosphoserine aminotransferase
M0ZP02	155;225;45	FFTSAGGLGK(1)PDIK	4.97	Phosphoglucomutase (alpha-D-glucose-1,6-bisphosphate-dependent)
M0ZR79	115;115;118	K(1)FDGQYWR	1.80	Threonine synthase
M0ZSA3	387;365	FGQYWIK(1)DGK	1.24	Monodehydroascorbate reductase
M0ZSA3	405;383	IVGAFLESGSPEENK(1)AIAK	2.19	Monodehydroascorbate reductase
M0ZTX6	108;108	ESSAK(1)MEVATDEQHTPIK	1.28	Inorganic diphosphatase
M0ZVM8	57;57;57	TAK(1)TIASPGR	1.24	Fructose-bisphosphate aldolase
M0ZVT8	311	AEVVSQDEK(1)ESGVR	1.74	Dehydroquinate synthase
M0ZWK0	61;61;36	ANSELSSHQK(1)K	1.37	Proteasome subunit alpha type
M0ZSD6	277;277	EESNDGSQK(1)ISGDQLK	1.91	Phosphopyruvate hydratase
M1A065	348	EVNDK(1)FLQAGLGR	1.33	Glyoxysomal fatty acid beta-oxidation multifunctional protein
M1A4U4	74;101	GFDK(1)AVWEVAEHK	1.59	Aldose-1-epimerase
M1ACS2	139;139;139	LQAGHK(1)YCLLGK	2.52	60S ribosomal protein L13a
M1ADQ4	188	LGTDEK(1)TFIR	1.65	Annexin
M1AKD9	50;50;45;45;50;45;45	ELISNSSDALDK(1)IR	2.98	Heat shock protein 83
M1AKD9	383;37;88;378	EMLQQNK(1)ILK	1.62	Heat shock protein 83
M1AKN6	70;70	LLDEHK(1)NPR	1.35	Phospholipase D
M1AZ78	173;206;81;166	K(1)FAIQNFVK	2.13	GrpE protein homolog
M1AXW2	201	RFGIAK(1)MEGK	1.27	3-hydroxyacyl-[acyl-carrier-protein] dehydratase
M1B054	160	TGGDK(1)YQWEIMR	1.89	Leucine--tRNA ligase
M1B054	144	VETEGNQPAPGGK(1)FK	1.22	Leucine--tRNA ligase
M1B583	481;397;481;481;481	AVENSPFLEK(1)LK	1.33	Heat shock cognate protein 80
M1B583	602;518;602;602;602	DSSMAGYMSSK(1)K	1.91	Heat shock cognate protein 80
M1B7A1	424;408;151	ETEK(1)NMSLR	1.38	ATSUG1
M1BMR5	95;138;130	FPSMTK(1)LR	1.25	Catechol oxidase
M1B7A1	98;98	VLVK(1)VHPEGK	1.34	ATSUG1
M1BAX6	438	AHSEK(1)SSVALVAQEK	1.24	30S ribosomal protein S15, chloroplastic

Accession	Positions	Acetyl (K) Probabilities	FC	Product
M1BCZ9	182	ALGMQVVK(1)K	1.23	Lactoylglutathione lyase
M1BJW9	374	LDMQDPNK(1)SVR	1.78	Glutamate--tRNA ligase
M1BK68	387;113	GGPNYQTGLAK(1)MR	1.38	ATP citrate synthase
M1BPZ5	400	SSIEK(1)GTGFVQK	1.43	Malate dehydrogenase
M1BRI8	136	PAAAAAPAK(1)K	1.33	Histone H1
M1BVA8	577	DK(1)DAVECCNK	2.34	Inosine monophosphate cyclohydrolase
M1BVW6	796;799;802;795;798	DK(1)EPLAAFDK	2.27	Lipoxygenase
M1BVW6	14;20	ELFGGHDDSK(1)K	2.40	Lipoxygenase
M1BVW6	814;817;653;817;820;817;813;817;800;816;817;817	LTDIEK(1)QIIQR	2.33	Lipoxygenase
M1BVW6	747;750;753;746;750;733;749	FMPEPGTPEYEELK(1)K	2.43	Lipoxygenase
M1BVW6	353;356;192;38;356;349;356;359;356;352;356;339;355;356;356	FPTPLVIK(1)DSK	2.43	Lipoxygenase
M1BZE9	235;249;271;278	LSDAPLAIVDK(1)R	1.42	Ribose-phosphate diphosphokinase
M1C004	200;200;200;200	FYK(1)EEEK	1.29	Phosphoglycerate kinase
M1C005	129;129;130	EEEK(1)NDPEFAK	1.26	Phosphoglycerate kinase
M1C1F1	257	LK(1)HTPPPHMIPPPPAPAK	3.85	Clathrin light chain
M1C415	156	LGYNGIK(1)VALPR	2.14	UDP-n-acteylglucosamine pyrophosphorylase
M1C6I0	247	GEVVAQLK(1)ASK	1.26	Asparagine--tRNA ligase
M1C8F8	194	NSVSTGEVYPLESEK(1)R	1.36	3-isopropylmalate dehydrogenase small subunit
M1C8Q0	167;54	LFEK(1)GGDWER	1.29	26S proteasome subunit RPN7
M1C9X0	392;93	IAEAIQK(1)K	1.30	Phosphopyruvate hydratase
Q9M4G4	569	LSK(1)MQEYTSR	1.47	Phosphoglucomutase, cytoplasmic
P43349	111;123	LEGEAQEAFK(1)K	1.34	Translationally-controlled tumor protein homolog
M1C9X0	392;93	IAEAIQK(1)K	1.30	Phosphopyruvate hydratase
P43349	111;123	LEGEAQEAFK(1)K	1.34	Translationally-controlled tumor protein homolog
P43349	84;96;58;84	LQEQPAFDK(1)K	1.23	Translationally-controlled tumor protein homolog
M1CDH5	409;443;409;409	EIHFLPFNPTDK(1)R	2.59	Plasma membrane ATPase
M1CGU5	49	SAITGGGGGSSASK(1)K	1.30	Salt tolerance protein 4
M1CQ92	485	VVVCQK(1)VGDASVVK	1.55	Pyruvate kinase
M1CQP6	306;306;214;306	ALQQSTLK(1)AWSGK	1.33	Fructose-bisphosphate aldolase
M1CQP6	38;38;26;38;38;38	GILAADESTGTIGK(1)R	1.23	Fructose-bisphosphate aldolase

Accession	Positions	Acetyl (K) Probabilities	FC	Product
M1CVZ7	390	AGGVLK(1)MIDEER	3.48	Delta-aminolevulinic acid dehydratase
M1CYB0	575	FMLAK(1)AAR	1.33	Protein tyrosine
M1CWD8	736	IVLDK(1)HEDTMSK	1.42	26S proteasome non-ATPase regulatory subunit 1 homolog
Q8GV42	60;60	DSNVPLDQNPK(1)LK	1.68	Hemoglobin
M1D6P6	136	DGLQNEK(1)TIIPTEVK	1.82	Hydroxymethylglutaryl-CoA lyase
Q0KIK9	851;916;44;915;916;916	EYPDFMDK(1)PPDK	1.69	RNA-dependent RNA polymerase
O22508	799;635;799;799;782;799;799	DK(1)EPLAAFDR	2.38	Probable linoleate 9S-lipoxygenase 8
O49150	453	INTTITK(1)SYASR	3.02	Lipoxygenase
P04045	749;364;757;758;352;273	AFATYVQAK(1)R	1.20	Alpha-1,4 glucan phosphorylase L-1 isozyme, chloroplastic/amyloplastic
P04045	287;157	AVAYDVPIPGYK(1)TR	1.36	Alpha-1,4 glucan phosphorylase L-1 isozyme, chloroplastic/amyloplastic
P04045	253;123	NDVSYPIK(1)FYGK	1.24	Alpha-1,4 glucan phosphorylase L-1 isozyme, chloroplastic/amyloplastic
Q2XPW5	40;41;41;41	APSVFEIK(1)NVGK	1.51	40S ribosomal protein S3a
P21343	405;390	DPHGNVQVAK(1)IETEK	1.21	Pyrophosphate--fructose 6-phosphate 1-phosphotransferase subunit beta
Q8LK04	263;263	EASYEDIK(1)AAIK	1.37	Glyceraldehyde-3-phosphate dehydrogenase
Q5CZ53	194;193	ASYIHHLDELK(1)K	2.75	Pom30 protein
Q9LM03	739;739	K(1)YTEVKPALQNMVSAAK	2.26	Methyltetrahydropteroyltriglutamate-homocysteine S-methyltransferase

Table S3. Differential acetylation sites between NAM treated dormancy and sprouting groups (NTDS).

Accession	Positions	Acetyl (K) Probabilities	FC	Product
M1CA90	371;371	DEGATILYGGDRPEHLK(1)K	0.78	Aminoaldehyde dehydrogenase 2
M0ZIL9	274	MVADGAVSCK(1)K	0.40	Nudix hydrolase domain-containing protein
M0ZSL5	322	IDYHTGVFASK(1)ITPAK	0.70	Dihydrolipoyl dehydrogenase
M0ZZV7	211;211;211	LVGVSEETTTGVK(1)R	0.61	Adenosylhomocysteinase
M1A0U1	158;158	QIFEADPNEQLK(1)K	0.79	GEM 5
M1A5P9	125;125;461;97	VWENFK(1)K	0.39	Cysteine proteinase inhibitor
M1AG69	26	PAAEK(1)APVAAAEK(1)SK	0.28	Histone H2B
M1B7W9	40	ERPVFSLSDDHAMSK(1)K	0.28	Protein SIEVE ELEMENT OCCLUSION B-like
M1C7V6	97;97;97;97	NVVLESK(1)YGAPK	0.82	Chaperonin-60 beta subunit
M1AY24	37	AEK(1)IILDYVHK	0.93	Peroxidase
M1CUT1	245	EVGGDLCFK(1)PSR	0.49	Anthocyanin acyltransferase
M1CV01	221	RLPGQQSGGEQFSLK(1)VPNNK	0.49	F17H15.1/F17H15.1
M1CXE9	136	HQLIK(1)IQR	0.81	Ornithine aminotransferase
M1D0M1	367	ENVLK(1)MEAQLAK	0.83	Pyruvate decarboxylase
M1CSJ6	490;490;484	SAK(1)YSLFDPCK	0.60	ADP,ATP carrier protein
M1BVA8	577	DK(1)DAVECCNK	1.66	Inosine monophosphate cyclohydrolase
M1C004	200;200;200;200	FYK(1)EEEK	1.48	Phosphoglycerate kinase
M1C415	156	LGYNGIK(1)VALPR	2.17	UDP-n-acteylglucosamine pyrophosphorylase
M1CQP6	103;103;11;103	EGGVLPGIK(1)VDK	1.26	Fructose-bisphosphate aldolase
M1D092	678	YQEIK(1)ENINEGLK	1.65	ALG2-interacting protein X
M1CQQ7	14;14;14;14;14	FSGAK(1)IYPGR	2.34	Ribosomal protein L24E
M1A4R1	281	DGSTVVK(1)SLETK	1.35	DNA-binding protein
M1AG47	7;7;7;7;7;7;7;7;7	AEK(1)K(1)PAEK	8.40	Histone H2B
M1AMB5	223;55	EGYEELENK(1)K	1.41	Cinnamoyl-CoA reductase
M1AXW2	171	QNFFFAGIDK(1)VR	1.59	3-hydroxyacyl-[acyl-carrier-protein] dehydratase
M1B069	158	SGIIVK(1)GIDIRPN	2.26	Tom
M1BUA0	233;146;234;233	VVGSEFVQK(1)YLGEGPR	1.86	26S protease regulatory subunit 6B
M1D0A6	277;228	EK(1)ATPDTIAK	1.29	Fructose-bisphosphate aldolase
M1D0M1	200	ELIAK(1)PIVQHSEIAK	4.38	Pyruvate decarboxylase
M1D5L7	82;105	EK(1)GIEVHER	4.61	Lactoylglutathione lyase
O24388	88;93;164	VGNENDIFK(1)IMK	1.31	Cysteine protease inhibitor 3 (Fragment)
Q3HVM3	21	FVAK(1)HGTAYYK	1.24	Hydrogen-transporting ATP synthase, rotational mechanism

Table S4. Differential acetylation proteins between natural dormancy and sprouting groups. (NPDS).

Accession	Fasta headers	Unique peptides	p-value	FC
A0A097H151	KTI-B protein	1	0.020872	0.430226
A0A7M1VJN5	Cruciferin PGCRURSE5-like variant Y1	2	0.023182	0.694589
M1AUK8	60S ribosomal protein L27	1	0.022713	0.739908
M0ZNL7	Glutathione transferase	2	0.006373	0.762248
M0ZR98	WD-repeat protein	2	0.025577	0.833031
M1A5P9	Cysteine proteinase inhibitor	2	0.044255	0.588840
M1BBE7	Major latex	2	0.032571	0.804495
M1AMY7	Cysteine protease inhibitor 1	4	0.035298	0.295794
M1AN26	Miraculin	4	0.034015	0.513862
M1CSU2	Transposase	2	0.039625	0.626488
M1BWL4	40S ribosomal protein S25	4	0.021654	0.758911
Q41415	Epoxide hydrolase	5	0.009509	0.808548
M1D2B0	Glutamate decarboxylase	4	0.041671	0.786380
M1D2B9	AGO1-1	10	0.042181	0.827095
M1A2Y4	Peroxidase	5	0.011720	0.810000
M1CW45	Catechol oxidase	1	0.042932	0.772213
O24383	Cysteine protease inhibitor 10 (Fragment)	6	0.024114	0.222849
O24388	Cysteine protease inhibitor 3 (Fragment)	5	0.012400	0.429397
Q8S393	Aspartic proteinase inhibitor P1E9	2	0.043730	0.332662
M0ZGB8	Reticulon-like protein	2	0.015715	1.544559
M0ZMF8	Phosphoserine aminotransferase	12	0.043718	1.776352
M0ZP02	phosphoglucomutase (alpha-D-glucose-1,6-bisphosphate-dependent)	5	0.040286	1.437712
M0ZRG6	Rubisco subunit binding-protein beta subunit	3	0.012076	1.760835
M0ZRH9	Small GTPase Rab2	1	0.033215	1.527331
M0ZSA3	Monodehydroascorbate reductase	19	0.012503	1.214782
M1A7M4	14-3-3 protein	5	0.024067	1.324260
M1AAF5	60s acidic ribosomal protein	2	0.017519	1.476902
M1AWB9	Glutamate-cysteine ligase, chloroplastic	7	5.08361E-05	1.739992
M1CPW5	Proteasome subunit alpha type	7	0.002846	1.218594
M1AD62	Neutral leucine aminopeptidase preprotein	20	0.008595	1.207800
M1BAR8	EMB2734	8	0.015127	1.272041
M1BJW9	Glutamate--tRNA ligase	5	0.023114	1.344444
M1BPZ5	Malate dehydrogenase	7	0.020299	1.482494
M1BTN1	Aspartate aminotransferase	6	0.015774	1.326047

Accession	Fasta headers	Unique peptides	p-value	FC
M1BXA1	[Acyl-carrier-protein] S-malonyltransferase	5	0.040506	1.749188
M1BYJ5	NADH: ubiquinone reductase (non-electrogenic)	2	0.036479	1.497363
M1C075	Heat shock cognate protein 80	1	0.004190	1.308330
M1C8Q0	26S proteasome subunit RPN7	8	0.020922	1.248136
M1C3S5	Pyruvate decarboxylase	5	0.045924	1.310607
M1C5X7	Oxoglutarate dehydrogenase (succinyl-transferring)	6	0.028599	1.336721
M1C9X0	Phosphopyruvate hydratase	7	0.004931	1.284523
M1CM34	Histone H2B	2	0.006028	3.106205
M1CTR3	Aconitate hydratase	17	0.020885	1.223268
M1D0M1	Pyruvate decarboxylase	20	0.013295	1.483899
P52402	Glucan endo-1,3-beta-glucosidase, basic isoform 3 (Fragment)	1	0.010158	3.061695
O49150	Lipoxygenase	3	0.012276	2.047479
Q6R2P7	14-3-3 protein	2	0.025319	1.341708
Q38JH1	GTP-binding nuclear protein	13	0.019603	1.272115
Q5CZ53	Pom30 protein	8	0.037187	1.324549
Q8H1X5	Allene-oxide cyclase	4	0.005999	1.379034

Table S5. Differential acetylation proteins between NAM treated dormancy and sprouting groups (NTDS).

Accession	Fasta headers	Unique peptides	*P*-value	FC
M1CQ13	Sucrose-phosphatase	6	0.02	0.81
M1AKE5	Aspartic protease inhibitor 5	2	0.02	0.56
M0ZHM4	Arginine--tRNA ligase	5	0.03	0.74
M0ZV99	Actin depolymerizing factor 3	3	0.03	0.74
M1A7Q8	Histone H2A	2	0.03	0.62
M1AE23	Protein Z	5	0.02	0.80
M1AS65	Transmembrane 9 superfamily member	2	0.05	0.76
M1AUM3	Cellulose synthase	4	0.04	0.71
M1AYK4	S-formylglutathione hydrolase	9	0.02	0.68
M1BFY0	Pre-mRNA-processing factor 19	4	0.02	0.75
M1C5J7	T-complex protein 1 subunit gamma	5	0.03	0.78
M1A2Y4	Peroxidase	5	0.01	0.8
M1CWA6	3-ketoacyl CoA thiolase 2	5	0.04	0.68
Q0KIK9	RNA-dependent RNA polymerase	3	0.02	0.65
M1A2F8	Glycosyltransferase	4	0.03	1.32
M1CCX0	Tyrosine--tRNA ligase	4	0.04	0.80
M0ZKH6	Glucose-6-Phosphate/phosphate translocator 1, chloroplast	4	0.04	1.36
M1BCP7	Thioredoxin-dependent peroxiredoxin	1	0.02	1.65
M1BPS2	Prefoldin subunit 4	2	0.03	1.50
M1D5L7	Lactoylglutathione lyase	3	0.02	2.05
Q307X7	50S ribosomal protein L22, chloroplastic	3	0.04	1.40

Table S6. Primers used for vector construction.

Primers	Sequence (5′–3′)	Purpose
pNC-AEnTopo-StPRX3-F<br>pNC-AEnTopo-StPRX3-R	atggctacatttgggtattt<br>ttaactattcacaaccgcacaa	Gateway
pNC-AEnTopo-StSIRT1-F<br>pNC-AEnTopo-StSIRT1-R	atgtctctgggttatgctgaaag<br>tcaccttacattaattacagcctctgg	Gateway
pNC-Cam1304SubC-StPRX3-F<br>pNC-Cam1304SubC-StPRX3-R	AGTGGTCTCTGTCCAGTCCTatggctacatttgggtattt<br>GGTCTCAGCAGACCACAAGTactattcacaaccgcac	Subcellular localisation
pNC-Cam1304SubC-StSIRT1-F<br>pNC-Cam1304SubC-StSIRT1-R	AGTGGTCTCTGTCCAGTCCTatgtctctgggttatgctgaaaa<br>GGTCTCAGCAGACCACAAGTccttacattaattacagctctggg t	Subcellular localisation
pNC-Cam2304-RNAi-StPRX3-F<br>pNC-Cam2304-RNAi-StPRX3-R	AGTGGTCTCTGTCCAGTCCTtagtgtctagcaatgtcaa<br>GGTCTCAGCAGACCACAAGTacaacacagatgcatcacat	RNAi
pNC-Cam2304-RNAi-StSIRT1-F<br>pNC-Cam2304-RNAi-StSIRT1-R	AGTGGTCTCTGTCCAGTCCTatggaacgatgcccttcttg<br>GGTCTCAGCAGACCACAAGTgctttcttgtccttgggagt	RNAi
pNC-Cam23FN-StPRX3-F<br>pNC-Cam23FN-StPRX3-R	AGTGGTCTCTGTCCAGTCCTatggctacatttgggtattt<br>GGTCTCAGCAGACCACAAGTttaactattcacaaccgcacaa	Overexpressi on
pNC-Cam23FN-StSIRT1-F<br>pNC-Cam23FN-StSIRT1-R	AGTGGTCTCTGTCCAGTCCTatgtctctgggttatgctgaaag<br>GGTCTCAGCAGACCACAAGTtcaccttacattaattacagcct ctgg	Overexpressi on
pGEX-4T-StPRX3-F<br>pGEX-4T-StPRX3-R	AGTGGTCTCTGTCCAGTCCTcaattacaacaaaacttcta<br>GGTCTCAGCAGACCACAAGTttaactattcacaaccgcacaa	Recombinan t expression
StPRX3^{K37Q}-F<br>StPRX3^{K37Q}-R	gtccaaagcagagcaaatcattttagactatgtccataaacacatt<br>ttgctctgcttttggacaactcttggcataga	Point mutation
StPRX3^{K37R}-F<br>StPRX3^{K37R}-R	caaagcagagaggatcattttagactatgtccataaacacatt<br>gatcctctctgcttttggacaactcttggcat	Point mutation

Note: Vectors used in LCA, BiFC, Y2H, Co-IP, and pull-down assays were constructed by recombination cloning without custom primers. Detailed procedures are provided in the Methods section.
